# The bioactive sphingolipid playbook. A primer for the uninitiated as well as sphingolipidologists

**DOI:** 10.1016/j.jlr.2025.100813

**Published:** 2025-04-18

**Authors:** Yusuf A. Hannun, Alfred H. Merrill, Chiara Luberto

**Affiliations:** 1 Departments of Biochemistry, Medicine, and the Stony Brook Cancer Center, Stony Brook University, Stony Brook, NY, USA; 2 School of Biological Sciences and the Petit Institute for Bioengineering and Biosciences, Georgia Institute of Technology, Atlanta, GA, USA; 3 Department of Physiology and Biophysics, and the Stony Brook Cancer Center, Stony Brook University, Stony Brook, NY, USA

**Keywords:** pathway analysis, targeted lipidomics, untargeted lipidomics

## Abstract

Sphingolipids and glycosphingolipids are among the most structurally diverse and complex compounds in the mammalian metabolome. They are well known to play important roles in biological architecture, cell-cell communication, and cellular regulation, and for many biological processes, multiple sphingolipids are involved. Thus, it is not surprising that untargeted genetic/transcriptomic/pharmacologic/metabolomic screens have uncovered changes in sphingolipids and sphingolipid genes/proteins while studying physiological and pathological processes. Consequently, with increasing frequency, both targeted and untargeted mass spectrometry methodologies are being used to conduct sphingolipidomic analyses. Interpretation of such large data sets and design of follow-up experiments can be daunting for investigators with limited expertise with sphingolipids (and sometimes even for someone well-versed in sphingolipidology). Therefore, this review gives an overview of essential elements of sphingolipid structure and analysis, metabolism, functions, and roles in disease and discusses some of the items to consider when interpreting lipidomics data and designing follow-up investigations.

## Graphical abstract

**Figure:**
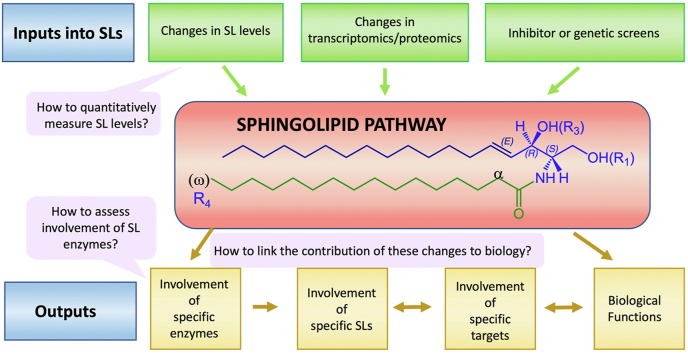

The study of sphingolipids has exploded over the past 4 decades with over 80,000 publications (PubMed search conducted on 4/8/2025 using terms: sphingolipid, glycosphingolipid, cerebroside, ganglioside, globoside, sulfatide, sphingomyelin, ceramide, sphingosine, sphingosine-1-phosphate, S1P, ceramide-1-phosphate, C1P) and with ∼90% of all the citations in PubMed in this field occurring during this period. In this “modern era,” the emphasis has also shifted because the majority of the earlier studies addressed aspects of the biochemistry and disease relevance of complex sphingolipids (including sphingomyelin, glycosphingolipids, and sulfatides), representing ∼66% of the citations between 1913 and 1983 whereas the more recent phase has focused heavily on the simpler compounds referred to as "bioactive" or "signaling" sphingolipids (ceramide, ceramide-1-phosphate (C1P), sphingosine and sphingosine-1-phosphate (S1P)), cited in more than 60% of the publications between 1984 and 2024 (Fig. 1). A new era of discoveries is beginning that encompasses all subcategories of sphingolipids and glycosphingolipids by utilizing new technologies for more comprehensive ("sphingolipidomic") analyses and the involvement of investigators with and without prior expertise in sphingolipid research.

**Fig. 1:**
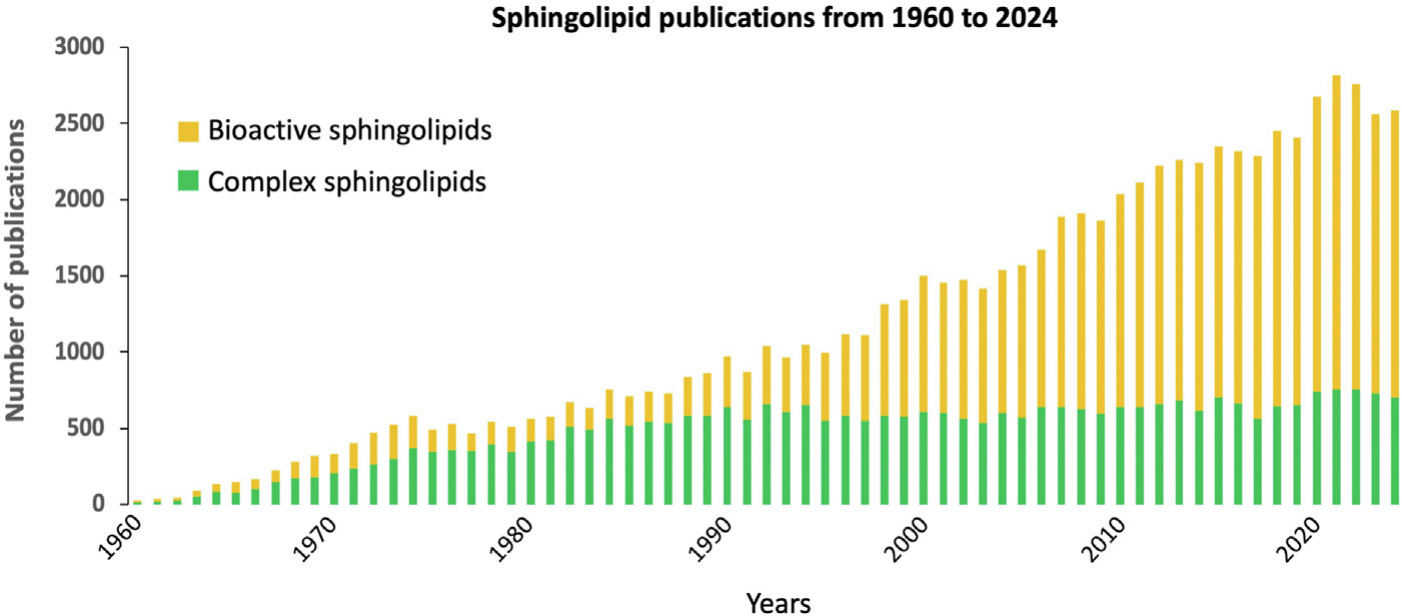
Number of PubMed citations tracking studies investigating sphingolipids over the years. A gradual increase in the total number of publications investigating sphingolipids can be observed from 1960 to 2024. Different profiles are apparent when using search terms that focus on complex sphingolipids (as defined below - green) versus bioactive sphingolipids (ceramide, sphingosine, S1P and C1P - yellow). Publications on complex sphingolipids were the vast majority in the early years while those citing bioactive sphingolipids became the majority in the last 25 years. PubMed searches were conducted on 4-8-2025. For complex sphingolipids in green, search included terms: glycosphingolipid OR sphingomyelin OR cerebroside OR sulfatide OR ganglioside OR globoside NOT ceramide NOT sphingosine NOT sphingosine-1-phosphate NOT S1P NOT ceramide-1-phosphate NOT C1P. For bioactive sphingolipids in yellow, search included terms: ceramide OR sphingosine OR sphingosine-1-phosphate OR S1P OR ceramide-1-phosphate OR C1P.

Complex sphingolipids play major roles in the structures of membranes, lipoproteins, and skin, and in cell regulation by defining specialized membrane domains (often referred to as "rafts"). They additionally have special roles in regulation of receptors and ion transporters, interactions between cells and the extracellular matrix, cell-cell communication, tissue development, cell differentiation, viral entry, bacterial toxin recognition, and other processes. Some glycosphingolipids serve as biomarkers for cancer and function as antigens targeted for immune-mediated pathology and therapy, e.g. CAR-T cells directed against GD2 in gliomas. This list of functions represents just the "tip of the iceberg" since only a fraction of mammalian glycosphingolipids, which have hundreds of distinct headgroups attached to numerous ceramide backbones (see section “Structures of sphingolipids”), has been studied in depth. As noted earlier, the simpler sphingolipids, including the metabolic intermediates, are now fully appreciated as important bioactive molecules in their own right. These play key roles in cell signaling, cell growth, cell differentiation, migration, inflammation, and many others.

Thus, sphingolipids exert and influence a myriad of cellular and pathobiological functions (see section “Functions of sphingolipids and mechanisms of action”). Indeed, based on this plethora of functions, we contend that most critical cell biological responses involve one or more bioactive sphingolipids at some juncture in the regulation of that specific response. This provocative hypothesis is being increasingly supported not only by studies focused on the analysis of specific sphingolipids but also by many discovery-type studies that apply “unbiased” (usually "omic") approaches to study diseases ranging from cardiovascular, metabolic, or neurodegenerative disorders to COVID-19 infections, as well as various cell processes. Many of these studies “stumble upon” remarkable changes in the sphingolipid pathway (defined here as sphingolipids, their enzymes, and sphingolipid-interacting proteins). Most non-lipidologists encounter sphingolipids either through 1) untargeted metabolomic/lipidomic approaches where they find statistically significant changes in levels of one or more sphingolipid, 2) untargeted transcriptomic and proteomic approaches that find statistically significant changes in the levels (mRNA or protein) of one or more enzymes/proteins of sphingolipid metabolism, and/or, 3) functional global screens using small molecule inhibitors or CRISPR/shRNA/siRNA pointing to sphingolipid genes being involved in a specific biological readout (Fig. 2). From such inroads into the world of sphingolipids, the initial and primary questions that arise pertain primarily to: 1) How to evaluate lipid changes and how to quantify them? 2) How to connect the involvement of a specific sphingolipid gene (or subset of genes) or enzymatic activity with alterations in the sphingolipidome, or possibly to the levels of specific bioactive sphingolipids? 3) What is the *functional* significance of these changes in lipids and enzymes? To these questions, the *sphingolipidologists* (and the intrepid non-lipidologist) would add: 4) What can we learn about the regulation, operation, and mechanisms, and spatial context of sphingolipids from these new associations and discoveries?

**Fig. 2:**
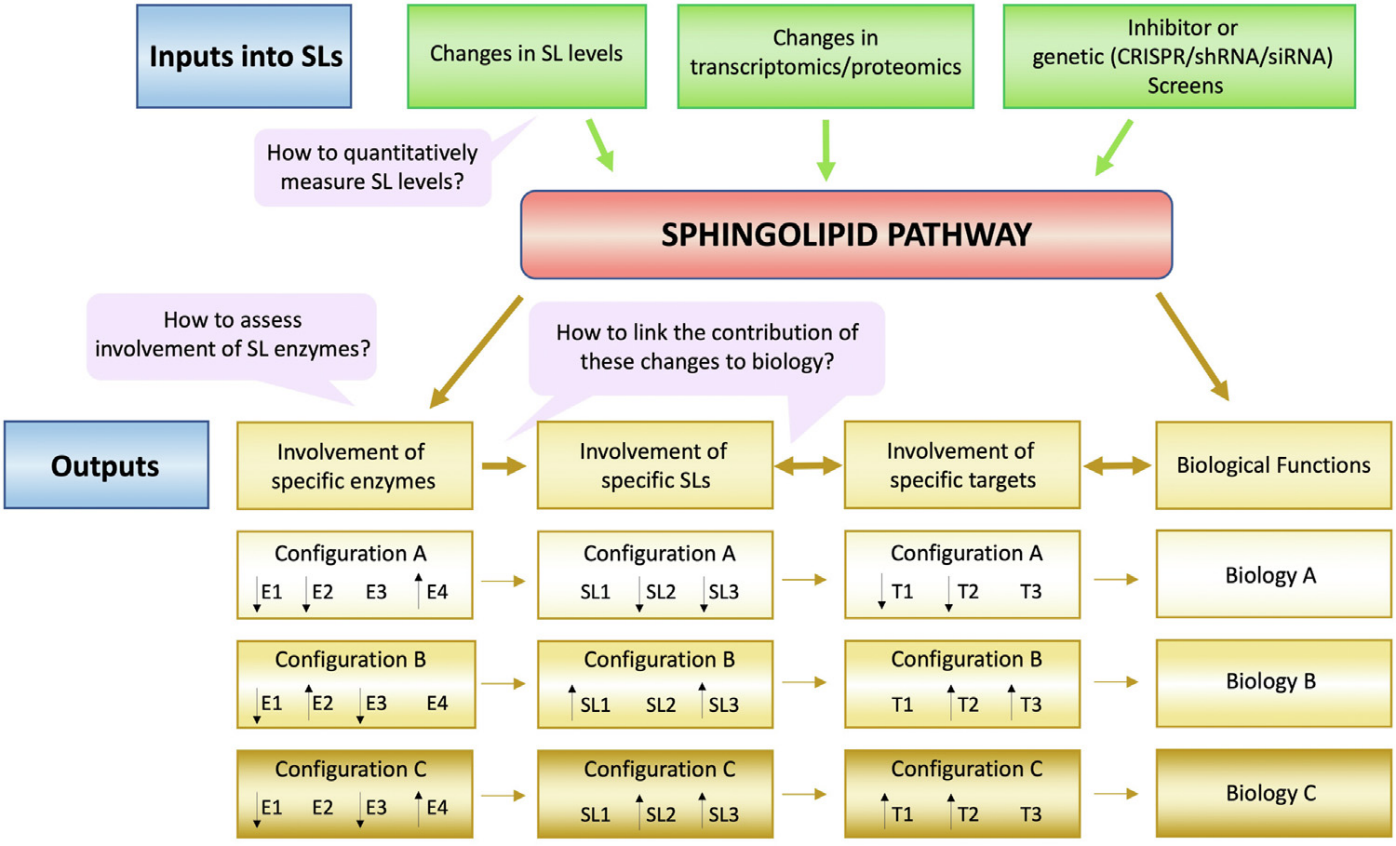
Paths into sphingolipid research and critical questions to make sense of the changes. Investigations using untargeted omics approaches may lead to uncover changes in sphingolipid levels or modulation of the expression of sphingolipid genes while genomic/inhibitor screens may also point to the functional importance of a specific sphingolipid enzyme. The immediate next steps require the researcher to biochemically connect the observed changes (the specific lipid profile with nodes/enzymes in the pathway) and establish when possible biochemical target(s). Because of the multiple levels of complexity that characterize sphingolipid metabolism including but not limited to having several bioactive molecules, different combinatorial outputs are expected depending on the reciprocal levels of the specific sphingolipid changes.

Unfortunately, pursuing studies on bioactive sphingolipids is fraught with many peculiarities and complexities that may appear daunting to the non-lipidologist (indeed, several aspects are difficult to navigate even for the well-versed sphingolipidologist). This review discusses some of the challenges of this universe and approaches to deal with them. Starting with the basics of sphingolipid structure, metabolism, functions, and roles in disease (these topics have been the subject of many other reviews), the review then discusses some approaches to navigate the intricacies of sphingolipid metabolism and function in one's own area of study.

## Structures of Sphingolipids

The first step in contemplating a sphingolipidomic data set is to appreciate the scope and complexity of the sphingolipidome. This will facilitate the selection of the appropriate lipidomic methodology(ies) and interpretation of the lipidomic results. This section describes the major subcategories of sphingolipids found in mammals and, in lesser detail, other organisms, including information about suggested nomenclatures and abbreviations (Box 1).

Box 1 **Nomenclature explained—LIPID MAPS conventions.**

Suggested nomenclature uses conventions that were originally recommended by the International Union of Pure and Applied Chemistry and the International Union of Biochemistry and Molecular Biology (IUPAC-IUBMB) (https://iubmb.qmul.ac.uk/) and recently expanded by LIPID MAPS for lipidomic data (1), as will be overviewed in this work. Other issues of nomenclature—namely what to do when there is ambiguity in the structure, such as the possibility of isomers or isobars—are discussed in the section “Functions of sphingolipids and mechanisms of action.”

**Fig. 3:**
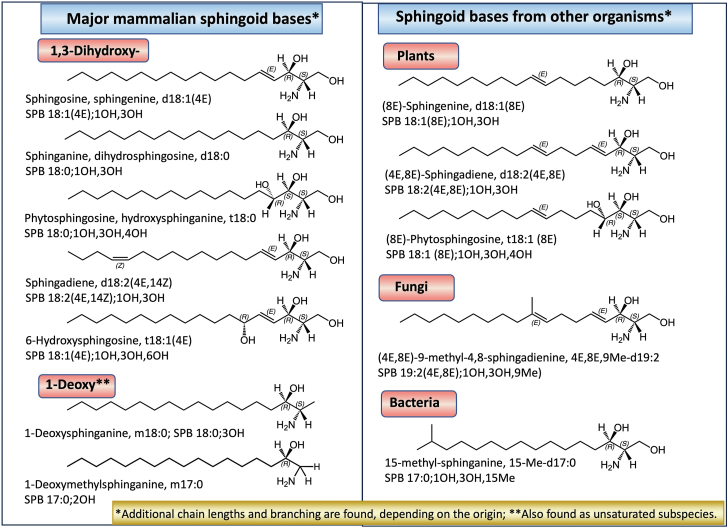
Subcategories of sphingoid bases produced by mammals and examples for other organisms. The major subcategories of sphingoid bases made by mammals are shown on the left and are subdivided into "1,3-dihydroxy" for those made using serine as the biosynthetic precursor and "1-deoxy" for those made from alanine or glycine (note: only the saturated 1-deoxy-sphingoid bases are shown--they are also found with double bonds). On the right are examples of additional structural variations seen in other organisms. Common names and abbreviations are given for these compounds. These are also found in other chain lengths and branching, as discussed in the text.

*Sphingosine* is defined as (2S,3R,4E)-2-amino-4-octadecene-1,3-diol by the IUPAC-IUBMB, with alternative names including (4E)-sphingenine and the abbreviation "d18:1(4E)". In the latter, "d" (for di-) refers to the two hydroxyl groups, “18” to the 18-carbon chain length and “:1” to the one *trans*-double bond, which is located between carbons 4,5 (4E). When full structures are known, LIPID MAPS recommends an abbreviation system that designates sphingoid bases as "SPB" with the number of carbons followed by number of double bonds, location and stereochemistry as above, followed by the locations of the hydroxyls; therefore, sphingosine is abbreviated SPB 18:1(4E);1OH,3OH (1). Figure 3 shows representative sphingoid bases of mammals and other organisms with their commonly used names and these two abbreviation systems; for example, phytosphingosine (also called 4-hydroxysphinganine) is abbreviated t18:0 by the older convention and SPB 18:0;1OH,3OH,4OH by the LIPID MAPS system.

*Ceramides* are the common names for N-acyl-sphingosines, *dihydroceramides* are N-acyl-sphinganines, *phytoceramides* are N-acyl-phytosphingosines (N-acyl-4-hydroxysphinganines), with designation of the specific amide-linked-fatty acyl chain by the chemical name for the fatty acid (for example, N-palmitoyl-sphingosine), or by designating the fatty acid by carbon number (such as C16-ceramide for N-palmitoyl-sphingosine) or by the LIPID MAPS nomenclature, which uses "Cer" as the category for all N-acyl-sphingoid bases, followed by specification of the sphingoid base type (using the abbreviation system above) followed by the fatty acid (by carbon number and number of double bonds), therefore, N-palmitoyl-sphingosine is abbreviated: Cer 18:1(4E);1OH,3OH/16:0 (1).

For **“Ceramide headgroup” derivatives**, the LIPID MAPS nomenclature identifies the subcategory of sphingolipid first, then the position where the characterizing derivative is located then the lipid backbone. Examples of LIPID MAPS nomenclature for some of these derivatives are indicated below (all with palmitate, C16:0, as the fatty acid):

***Ceramide 1-phosphates*** are commonly abbreviated Cer1P or C1P; the LIPID MAPS abbreviation is "CerP(1) 18:1(E);3OH/16:0" (note that "1OH" has been removed because the hydroxyl at carbon #1 is attached to phosphate).

***Ceramide 1-phosphocholines*** are commonly called sphingomyelins (SM); the LIPID MAPS abbreviation is "SM(1) 18:1(4E);3OH/16:0."

***Ceramide 1-phosphoethanolamines*** are sometimes abbreviated CerPE or CPE; the LIPID MAPS nomenclature is "PE-Cer(1) 18:1(4E);3OH/16:0."

***Ceramide 1,3-cyclic phosphates*** are abbreviated "CerP(1, 3) 18:1(4E)/16:0" by the LIPID MAPS system.

***Glucosylceramides*** are commonly abbreviated GlcCer; the LIPID MAPS nomenclature is "GlcCer(1) 18:1(4E);3OH/16:0."

***Galactosylceramides are*** commonly abbreviated GalCer; the LIPID MAPS nomenclature is "GalCer(1) 18:1(4E);3OH/16:0."

***Glycosphingolipids***. The annotation system that is used for the sugar moieties in complex glycosphingolipids is the same as that followed by glycan science (https://www.ncbi.nlm.nih.gov/glycans) and, like the similar IUPAC-IUB guidelines, is intended to clarify any ambiguities among different glycans that have the same sugar composition but differ in linkages. For example, GM1a (in the lower right of Fig. 5) would be described as Neu5Acα2-3(Galβ1-3GalNAcβ1-4)Galβ1-4Glcβ1Cer (plus designation of the lipid backbone using the LIPID MAPS abbreviation system). Another naming system builds on the root structure with designation of the location of additional substituents, such as the Neu5NAc on the ganglio- (Gg) glycan of ganglioside GM1a using Roman numerals and Arabic superscripts to designate the hydroxyl to which the Neu5NAc is linked. By this system, GM1a is described as II^3^-α-Neu5NAcGg_4_Cer (which would be read “II^3^-α-N-acetylneuraminosyl-gangliotetraosylCer”). This naming can also add any additional modifications, such as 9-O-acetylation of sialic acid. Some glycosphingolipids are still most often referred to by historic names, such as the Lewis blood group antigens (an example is Fucosyl-GM1 shown in Fig. 5). The shapes and colors of the glycans shown in Figure 5 also follow the convention of the glycan community.

### The sphingoid bases

Sphingoid bases are the backbones for all sphingolipids and show considerable diversity within and across organisms (Fig. 3) (2, 4). Sphingosine is the prototypic sphingoid base and is specifically defined as (2S,3R,4E)-2-amino-4-octadecene-1,3-diol by the IUPAC-IUBMB. There are hundreds of variations of this structure in nature (2), and the structures, commonly used names, and abbreviations for the sphingoid bases made by mammals plus a few for other organisms are shown in Figure 3. Note that the sphingoid bases are shown as the neutral amines, but a fraction will be protonated at physiologic pH due to having a pKa of approximately 7 (3, 5).

Mammalian sphingolipids are comprised of varying proportions of the sphingoid bases in the left panel of Figure 3: sphingosines, sphinganines (sometimes referred to as dihydrosphingosines) and (4E,14Z)-sphingadienes depending on the tissues (6), plus phytosphingosines (4-hydroxysphinganines) for epithelial tissues such as intestine and skin (7) (the latter additionally has 6-hydroxysphingosines (8)). Not shown are 3-ketosphinganines, which are intermediates of de novo biosynthesis of sphingoid bases that, differently from yeast, occur in trace amounts in mammals except in unusual circumstances, such as genetic defects in the 3-keto reductase (9). The most recently discovered sphingoid base subcategories that are produced by mammals and other organisms are sometimes referred to as "atypical" because they lack the hydroxyl at carbon 1 (1-deoxysphingoid bases) or the entire hydroxylmethyl moiety at that position (1-deoxymethylsphingoid bases) (Fig. 3) (10, 11) (in our discussion, we will not use "atypical" because some other articles have used this term in reference to miscellaneous uncommon sphingolipids and will refer to these sphingolipids as “non-canonical”). While 1-deoxy-sphingolipids can be found in almost every mammalian tissue (and sometimes in high amounts in cells in culture) (12, 13), little is known about their physiologic functions, but recent studies suggest roles in modulation of orphan nuclear hormone receptors (14) and in feedback regulation of serine metabolism (15). Other recent studies are implicating the deoxysphingolipids in many disease states, including neuropathies and macular degeneration (11). Therefore, it is advisable to include 1-deoxy-sphingoid bases in sphingolipidomic studies.

The chain length of the sphingoid bases of most mammalian sphingolipids is 18-carbons, as shown in Figure 3, but other chain lengths from 12- to 26-carbons (as well as occasional iso- and ante-iso branching) have been measured (2). Examples are the d16-sphingoid bases in myocardium (16) and d20-in distinct anatomical regions of the brain (17). It is noteworthy that abnormal levels of these sphingosine variants appear to have deleterious consequences (16, 18). In contrast, other organisms (such as insects) frequently have d14- and d16-sphingoid bases (2). Odd-carbon-chain-length sphingoid bases can have either linear or branched chains (such as the fungal (4E,8E)-9-methyl-4,8-sphingadiene shown in Fig. 3) but are thought to be relatively uncommon in mammals except under special conditions (19). However, this might be due to the lack of studies that have attempted to find them because d17- and d19-sphingoid bases are detectable in small amounts when looked for (20), and ante-iso-sphingosine (ie, 16-methyl-sphingosine) has been found to be produced by mammalian SPTLC3, one of the subunits that constitute the enzyme regulating the first step of de novo sphingolipid synthesis (21). Odd-chain length sphingoid bases are most common in bacteria and fungi (2) (see examples in Fig. 3), and food is another source for them for humans (for example, (4E,8E)-9-methyl-4,8-sphingadiene is in *Koji*, a fermented food prepared using *Aspergillus oryzae* (22)).

Although mammals produce sphingoid bases with double bonds only in a few positions (4E and 14Z) (23), other organisms produce sphingoid bases with double bonds on other carbons (2) (see Fig. 3, right side). These additional double bond positions (and other structural variants) should be kept in mind during lipidomic analyses of human tissues because they can be taken up to some extent from food and intestinal microflora (24, 25, 26, 27, 28).

For additional information about structural variations, including those found in other organisms, see (2, 4, 28, 29, 30, 31, 32, 33).

### Sphingoid base phosphates and other derivatives of sphingoid bases

**Fig. 4:**
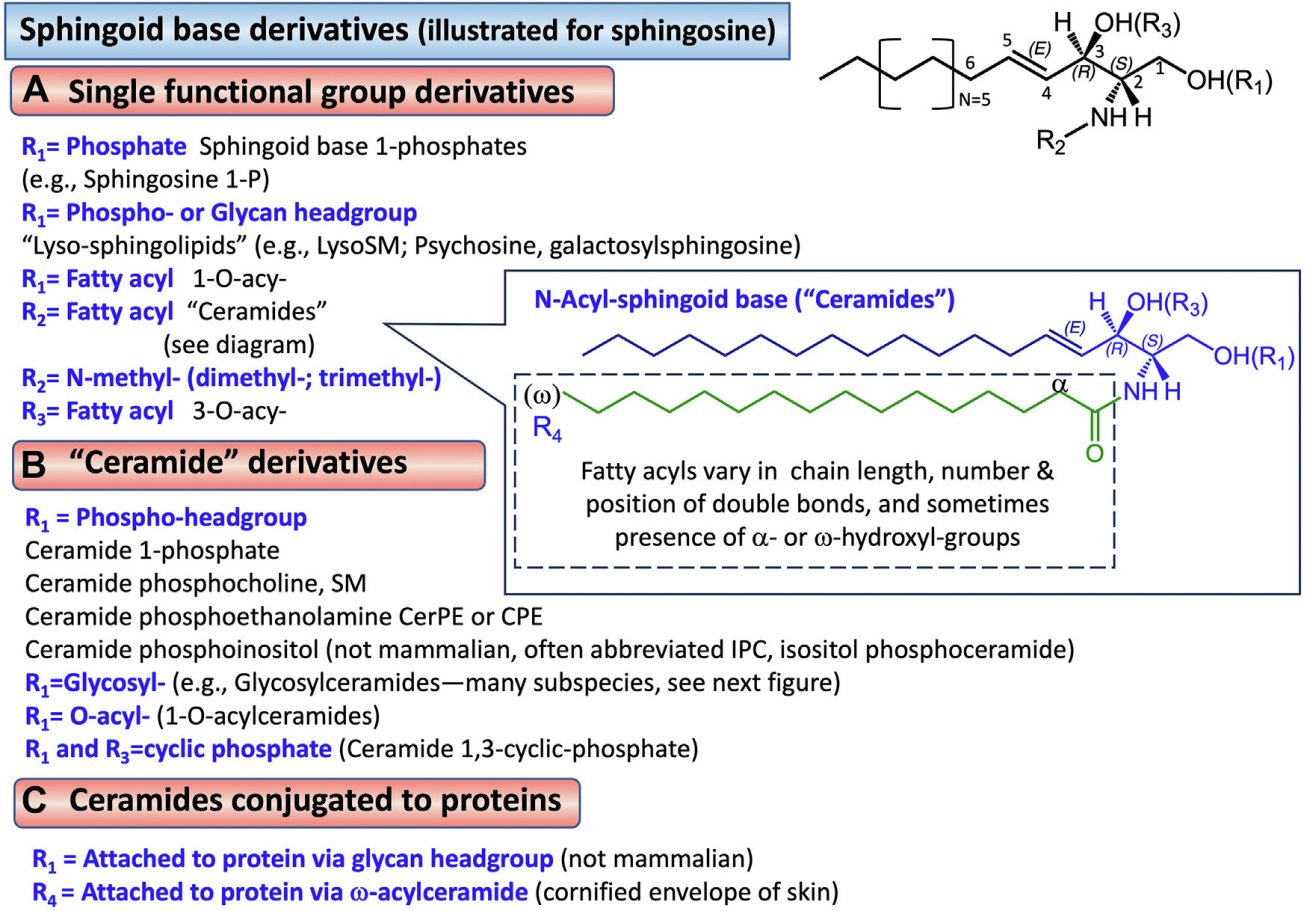
Major subcategories of sphingolipids made from sphingoid bases. A: A prototype sphingoid base shown on the right can be modified on any of the derivatizable groups--i.e., the 1-hydroxyl (R1) for sphingoid base 1-phosphates, lyso-sphingomyelin and lyso-glycosphingolipids; the amino moiety (R2) for ceramides and N-methyl-sphingoid bases; and acylation on either the R1 or R3 hydroxyls. B: Summary of the various types of derivatives of the 1- and/or 3-hydroxyls of ceramides. For more complex glycosphingolipids, see Figure 4. C: Covalent attachment of ceramides and more complex sphingolipids to proteins.

The single functional group derivatives occur on either the 1-hydroxyl or 2-amino moieties (R1 and R2, respectively, in Fig. 4). The moieties found on the 1-hydroxyl are phosphate (for sphingosine 1-phosphate, sphinganine 1-phosphate, etc.) and more complex headgroups for the so-called "lyso-" sphingolipids, such as sphingosylphosphocholine (lysosphingomyelin) (34), glucosyl- and galactosyl-sphingosines (the latter is also called psychosine), and other de-acylated versions of more complex sphingolipids (35). The 2-amino derivatives are predominantly amide-linked fatty acids to constitute "ceramides" i.e., N-acyl-sphingoid bases (see below). An additional type of sphingoid base modification is methylation of either the 1-hydroxyl- or the 2-amino groups. These can appear as artifacts during the extraction and handling of sphingoid bases (36, 37), but they are also formed naturally by mammals (38, 39).

### Ceramides

The term "ceramides" has been used in the literature to reflect specifically N-acyl-sphingosines, "dihydroceramides" for N-acyl-sphinganines, "phytoceramides" for N-acyl-phytosphingosine (N-acyl-4-hydroxysphinganines), etc., but the term ceramides has also been used collectively for all these subclasses. In mammals, the fatty acyl chains of ceramides have been found to range from 2 carbons (ie, acetyl) (40) to more than 36 carbon atoms, although most of mammalian tissues have sphingolipids with chain lengths of 14–26 carbons (noteworthy exceptions being skin and testis, which have longer chain lengths) (8, 41). The fatty acids are primarily saturated and monounsaturated, but there are many instances where two or more double bonds are present (ceramides in testis, for example, have very-long chain polyunsaturated fatty acids) (41). Another feature of the fatty acids of mammalian ceramides is that they sometimes have an α- or ω-hydroxyl group. The latter is specifically found in skin ceramides (8) where it is often esterified to other long-chain fatty acids, especially linoleic acid, to form so-called ω-acylceramides, which stabilize the lipid lamellae as well as become covalently attached to cornified envelope proteins (42). There are so many varieties of skin ceramides that another series of abbreviations is often used in describing them (8, 42).

### Phosphoceramides and sphingomyelins

Phosphosphingolipids produced by mammals are 1-OH derivatives: 1) ceramide 1-phosphates, which are commonly abbreviated Cer1P or C1P; 2) ceramide 1-phosphocholines, commonly called sphingomyelins (SM) with the assumption that the user knows the headgroup is phosphocholine--which is also assumed by the LIPID MAPS abbreviation (Box 1); and 3) ceramide 1-phosphoethanolamines (CerPE or CPE). These phosphosphingolipids are also found in many other organisms, as is the additional subcategory of the ceramide phosphoinositols (or more often called inositol phosphorylceramides (IPC)), which are present in fungi (30, 43), plants (31) and protozoa (44, 45, 46), and are often elaborated to more complex glycans (43, 47). Mammals do not appear to make IPC although they make glycosylphosphatidylinositol-anchored proteins (48). An additional type of phosphoceramide is produced when SMs or CerPEs are cleaved to ceramide 1,3-cyclic phosphates by a category of phospholipase D found in the venom of some spiders as well as in some bacteria and fungi (49).

### Glycosylceramides

**Fig. 5:**
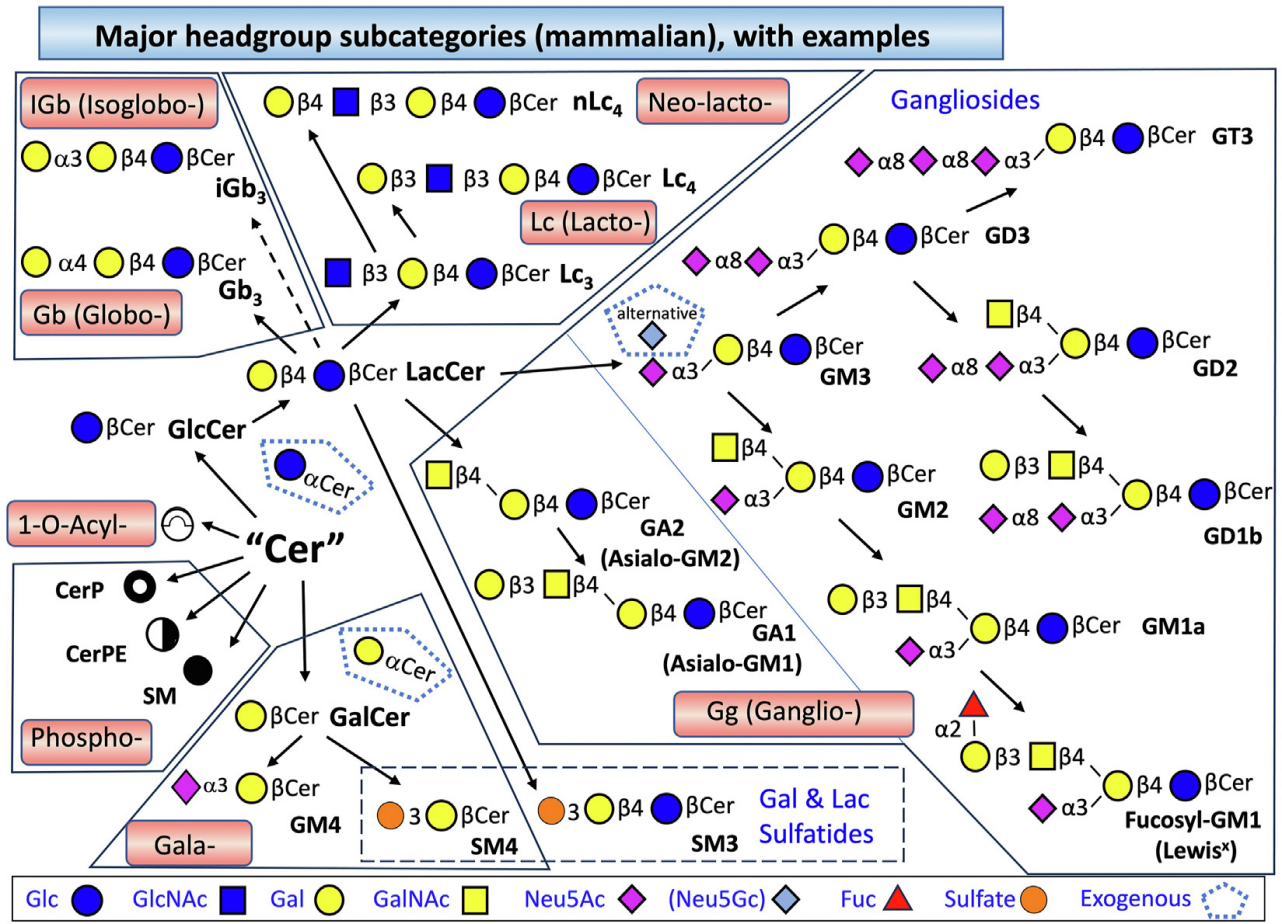
Major subcategories of complex (glyco)sphingolipids produced by mammals from ceramides, organized by pathway of origin. Starting with ceramides (Cer) to the left of the diagram, each arrow depicts a headgroup addition by enzymes named in the text or cited references to produce the shown subcategories of complex sphingolipids, named (clockwise); GlucosylCer (GlcCer) and more complex glycosphingolipids as described below; GalactosylCer (GalCer) and others; the phosphosphingolipids Sphingomyelin (SM), Ceramide phosphoethanolamine (CerPE) and Ceramide 1-Phosphate (Cer1P); and 1-O-Acyl-Cer. For the more complex glycosphingolipids, the position and type of linkage is also given (*eg*, formation of a glycosidic bond between Gal and the 4-hydroxyl- of GlcCer in beta-linkage produces Galβ4GlcβCer, which has the common name LactosylCer, LacCer). Depending on the next addition, the products are subcategorized as shown in the various polygons. LacCer can also undergo sulfation to "SM3) as shown at the bottom (note: the abbreviation does not designate it is a sphingomyelin), as shown at the bottom, and subcategorized as a "sulfatide," with other sulfated glycosphingolipids, such as sulfated GalCer, SM4. In the "Ganglio" subcategory, "Gangliosides" are generally named as compounds with one or more sialic acids (mainly N-acetylneuraminic acid for humans). Also given are some of the common names for these complex glycosphingolipids (*eg*, gangliosides GM3, GD2, etc.). When fucose is added to a glycosphingolipid, the term "Fucosyl-" can be added to the parent glycosphingolipid (as shown for Fucosyl-GM1 in the lower right corner), or the compound might be referred to by a historically assigned name, such as Lewis^x^ in this example. The symbols for these carbohydrates are given at the bottom (glucosyl-, Glc; N-acetylglucosamine, GlcNAc; galactosyl-, Gal; N-acetylgalactosylamine, GalNAc; N-acetylneuraminic acid, NeuAc; and Fucosyl-, Fuc) and have been adopted by the international glycan community; others (such as for SM, CerPE, etc.) are for illustrative purposes in this review.

This class represents a very large family of compounds that mammals produce by adding either a glucose (Glc) or galactose (Gal) to the 1-hydroxyl group of ceramides in β-glycosidic linkage to form Glucosylceramide (GlcCer) and Galactosylceramide (GalCer), respectively (50, 51, 52). This is followed by additional substituents that include both α- and β-glycosidic linkages to make the more complex glycosphingolipids shown in Figure 5. The compounds in this figure were chosen as representatives of each major subcategory, and the key at the bottom summarizes the carbohydrates (plus sulfate for sulfatides) that are found in mammalian glycosphingolipids. The symbols for each carbohydrate follow the convention of the glycan community to use both color and shape to designate the compounds (https://www.ncbi.nlm.nih.gov/glycans/snfg.html) (53). Other organisms utilize many of these and additional carbohydrates, such as mannose and glucuronic acid, to make glycosphingolipids (50, 51, 52).

The dashed pentagon around three special cases indicates glycosphingolipids that might arise from an exogenous source, or at least are comprised of components that are from exogenous sources. Examples of glycosphingolipids found in mammals after uptake from exogenous sources are: α-anomers of GlcCer and GalCer (54) apparently produced by bacteria in the intestinal microflora (55); some plant GlcCers that are taken up intact from the diet (26); and, gangliosides containing N-glycolylneuraminic acid (Neu5Gc) instead of the endogenously synthesized sialic acid, N-acetylneuraminic acid (Neu5Ac) from utilization of exogenous Neu5Gc (56).

It should be mentioned here that much of the lipidomics literature reports data for "hexosylceramides" (HexCer), which are the sum of GlcCer and GalCer, instead of the individual subspecies due to the lack of resolution of these isomers by the analytical method adopted (this applies to many of the untargeted and targeted lipidomics analyses). However, since these constitute two entirely distinct and important branches of glycosphingolipid metabolism (see Fig. 5) and there are methods to separate them by liquid chromatography (LC) (57, 58, 59) or with differential ion mobility-mass spectrometry (60), they should be distinguished in sphingolipidomic analysis.

The nomenclature for more complex glycosphingolipids uses the subcategory names that are in current practice in glycan science (https://www.ncbi.nlm.nih.gov/glycans/) with specification of the lipid moiety as described above. The glycan subcategories are illustrated in Figure 5 by the ways certain molecules are enclosed by the irregularly shaped lines. After GlcCer is metabolized to LacCer, the next carbohydrates that are added determine whether the downstream glycosphingolipids are in the lacto-series (abbreviated Lc) or their isomeric series neo-lacto (nLc), globo- (Gb) or its isomeric series isoglobo (iGb) (which is not produced by humans), or ganglio- (Gg). These are referred to as the "root structures" (https://www.ncbi.nlm.nih.gov/books/NBK579905/). There are also nomenclatures that overlap with these designations, such as the "gangliosides," which connote the presence of a sialic acid (typically Neu5Ac but sometimes Neu5Gc, as discussed above), and the sulfated glycosphingolipids, termed "sulfatides." See Box 1 for further nomenclature.

Only a few of the lipidomic studies to date have attempted to include the more complex sphingolipids in the analysis because of the challenges in identifying and quantifying these compounds; however, tools for such analysis are becoming available. These compounds are clearly very important, so they will undoubtedly appear with increasing frequency. Those who need to sort through the lipidomic data for interpretation of the findings in the context of structure and pathway maps can utilize a number of tools, such as the LIPID MAPS Mass Spectroscopy Analysis Tools (https://lipidmaps.org/resources/tools/ms), the KEGG Pathway maps for lacto- and neolacto-series (https://www.kegg.jp/kegg-bin/show_pathway?map00601), globo and isoglobo-series (https://www.kegg.jp/kegg-bin/show_pathway?map00603) and ganglio-series (https://www.kegg.jp/kegg-bin/show_pathway?map00604) glycosphingolipids, and the WikiPathways Project database (61) available on the LIPID MAPS website (https://lipidmaps.org/resources/pathways).

### O-acyl-ceramides

*1-O-Acylceramides* have a fatty acyl group attached to the 1-carbon of the sphingoid base backbone. They are primarily found in skin, and the major subspecies in human stratum corneum have lignoceric acid (24:0) or palmitic acid (16:0) as the fatty acyls at position 1, and sphingosine (d18:1(4E)) plus either palmitic acid (16:0) or α-hydroxypalmitic acid (h16:0) as the N-linked fatty acyls of the ceramide backbones (62). They were estimated to be present in substantial amounts (nearly a third) compared to the corresponding unmodified parent ceramides. More recently, 1-O-acylceramides have also been measured in lipid droplets (63).

*3-O-Acetyl-ceramides* have an acetyl group on the 3-hydroxyl of the sphingoid base and were originally noticed as fast-migrating cerebrosides (3-O-acetyl-GalCer) that are enriched in myelin and myelinated nerve fibers (64). Other types of fast-migrating cerebrosides were found with acetyl groups on some of the galactose hydroxyls.

It is noteworthy that these O-acyl-sphingolipids (as well as gangliosides that have O-acetylated sialic acid, such as 9-O-Ac-GD3 (65)) are labile to the mild alkali treatment that is often used to remove interfering glycerolipids during the extraction and analysis of sphingolipids. Thus, they and additional O-acylated sphingolipids might be encountered more often when lipidomic analyses are conducted in situ or with samples that have not been treated with acid or base during the extraction.

### Proteins conjugated to ceramides

Two types of sphingolipids have been found to be conjugated with proteins (Fig. 4).

i) *Inositol phosphorylceramide-glycan anchored proteins*, found on the membranes of yeast (66) and the surface membrane of the protozoa *Trypanosoma cruzi* (44). These generally have a core glycan (mannose(α1-2)mannose(α1-6)mannose(α1-4)glucosamine) that is attached to the myo-inositol of IPC, and a linker (such as ethanolamine phosphate or 2-aminoethylphosphonate) that is attached to the glycan core via its phosphate and to the protein via its amine (44, 47, 67, 68).
ii) ω-*Acylceramide-linked proteins*, found in the cornified envelope of skin. The most recent model for their structure is for the linoleic acid that is linked to the ω-hydroxyl of acylceramides to be converted into an epoxy-enone that is covalently bound to the cornified envelope proteins by a Michael addition reaction or Schiff base formation (42).

## Sphingolipid Biosynthesis and Turnover

**Fig. 6:**
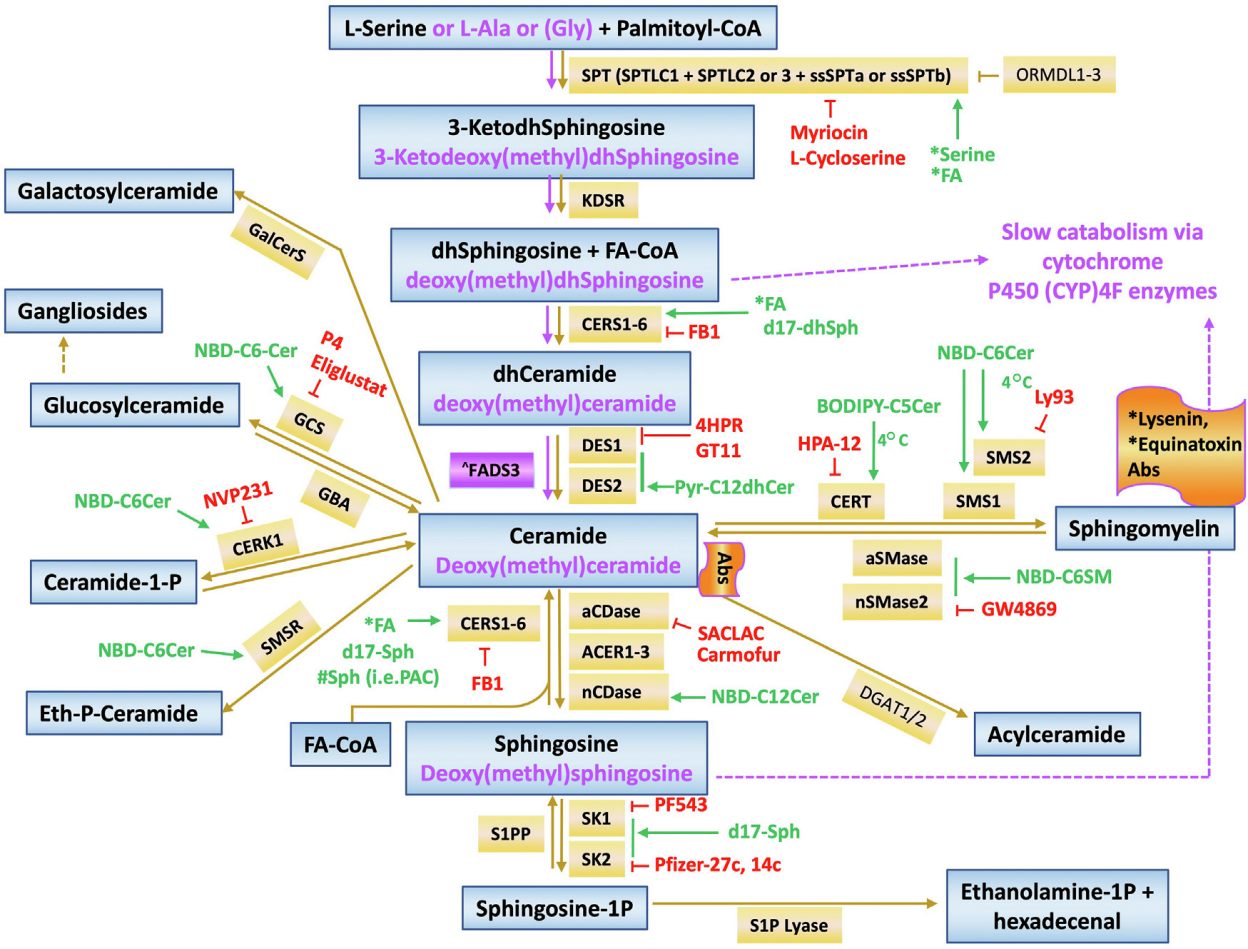
Schematic of the sphingolipid metabolic pathway and biochemical tools for its investigation. Synthesis of canonical sphingolipids (indicated in black) starts with the condensation of L-serine and palmitoyl-CoA by SPT. L-alanine (L-Ala) or glycine (Gly) can also be utilized instead of serine to produce deoxysphingolipids (deoxysphingolipids are reported in light pink). In red are inhibitors available to researchers. Whereas many additional inhibitors have also been reported in the literature, in this Figure we are indicating the more specific ones. In green are the various precursors that can be utilized to study flux through the pathway or through a specific metabolic node. ∗: indicates that the molecule is labeled (lipid precursors can be traced isotopically, radioactively or complexed to a fluorescent moiety; toxins may be fused to small fluorescent reporter proteins such as green or red fluorescent proteins). #Sph: these are bi- or tri-functionalized sphingolipid analogs. ˆFADS3: This enzyme introduces a Δ14,15 *cis* double bond onto deoxy(methyl)DHCer or deoxy(methyl)dhsphingosine to form deoxy(methyl)ceramide or deoxy(methyl)sphingosine, respectively. For canonical sphingolipids, DES introduces a Δ4,5 *cis* double bond onto dhCeramide to form ceramide. Abs: antibodies.

**Fig. 7:**
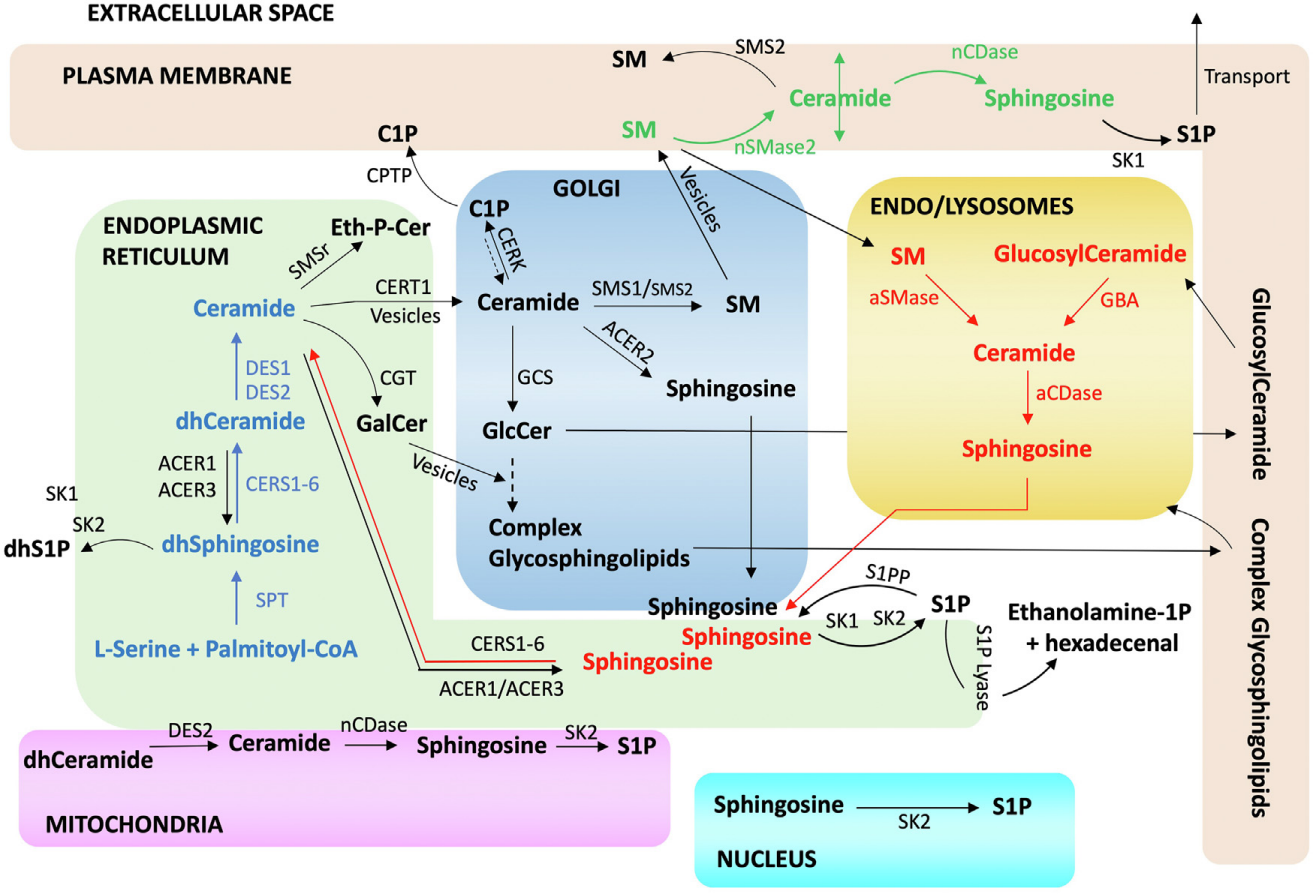
Schematic of metabolic building blocks within the sphingolipid pathway and their subcellular localization. The sphingolipid pathway can be schematically organized in different blocks which represent both metabolic nodes and subcellular compartments. The first steps of the de novo sphingolipid metabolism occur in the endoplasmic reticulum (ER) to form ceramide (reactions in blue). Ceramide then gets transported to the Golgi via CERT1 or vesicular trafficking. In the Golgi, complex sphingolipids are produced (reactions in black) and are then transported to the plasma membrane. In the plasma membrane, ceramide can be converted into either sphingosine or sphingomyelin (SM) and vice versa (reactions in green). Sphingosine in the plasma membrane can also be converted into sphingosine-1-phosphate (S1P) by plasma membrane-associated sphingosine kinase 1 (SK1). S1P at the plasma membrane can then be transported in the extracellular space by dedicated proteins such as Spinster homologue 2. From the plasma membrane, sphingolipids are recycled into the endo/lysosomal pathway where they are progressively broken down to form sphingosine (reactions in red). Lysosomal sphingosine can either 1) re-enter sphingolipid synthesis via conversion into ceramide (and eventually complex sphingolipids) or 2) be converted into (S1P). S1P can then be irreversibly broken down by the S1P lyase into ethanolamine-1-phosphate and hexadecenal with the only known exit reaction from the sphingolipid pathway. In addition to ER, Golgi, PM and lysosomes, evidence indicate the existence of discreet sphingolipid metabolic nodes in the mitochondria and nuclei. Abbreviations used are defined in the main text. CPTP: the recently identified C1P transfer protein. The dashed arrow from C1P to ceramide indicate a step not well characterized.

The committed synthesis of sphingolipids is initiated in the endoplasmic reticulum (ER) by the action of serine palmitoyl transferase (SPT) which condenses serine (but also alanine or glycine) and a fatty acid, primarily palmitoyl CoA (which has 16 carbons, but also stearoyl CoA with 18 carbons and myristoyl CoA with 14 carbons), leading to the production of 3-ketodihydrosphingosine(s) (Fig. 6). This kick-starts the de novo pathway of sphingolipid synthesis. Reduction of 3-ketodihydrosphingosine by a specific reductase generates dihydrosphingosine(s). This is then acylated by one of 6 distinct (dihydro)ceramide synthases (CerSs) to form dihydroceramides. Following, dihydroceramide desaturases (DES1/2) act on dihydroceramides to form ceramides and phytoceramides (51, 69, 70, 71) (Fig. 6). All these steps occur in the ER (Fig. 7), with the addition of the synthesis of 1-acylceramides by the actions of DGAT1/2, as recently shown by the Obeid group (63). Ceramides are transported from the ER to the Golgi where they serve as precursors to SMs through action of SM synthases (SMSs) or to GlcCer through action of glucosylceramide synthase (GCS). Transfer to the Golgi occurs by two mechanisms: vesicular trafficking couples the transported ceramides to the synthesis of GlcCer whereas transport via the direct action of the ceramide transfer protein (CERT) couples them to SM synthesis (72) and possibly also to synthesis of C1P by ceramide kinase (CERK) (73). GlcCer reaches the Trans Golgi Network (TGN) passing through the Golgi cisternae (for Gg sphingolipid synthesis) or transported by the FAPP2 transfer protein (for Gb sphingolipid synthesis) (Fig. 5) (51, 74, 75, 76, 77). In the TGN, synthesis of glycosphingolipids develops by the sequential addition of sugar subunits and sialic acid residues (the latter characterize the Gg subgroup of complex glycosphingolipids) (78, 79). GalCer instead is formed in the lumen of the ER by the Ceramide Galactosyltransferase (CGT) (which is widespread but most active in neural tissue) and, once transported to the TGN, it serves as the foundation molecule for a much smaller group of glycosphingolipids, which includes the sulfatides. SM and the bulk of glycosphingolipids then reach the plasma membrane via Golgi-derived vesicles (76); glycosphingolipids and C1P may reach this compartment also with the assistance of the glycosphingolipid transfer protein, GLTP (80) and the C1P transfer protein (CPTP) (81), respectively. From the plasma membrane, sphingolipids can be internalized in the endosomal system. They then reach the lysosomes where they undergo degradation through the action of various glycosidases, hydrolases, and acid sphingomyelinase (aSMase) (82, 83). This results in the formation of ceramide which is then further degraded by acid ceramidase (aCDase) to sphingosine. Sphingosine, while favored to partition inside the lysosome because of its positive charge, can efflux from lysosomes, possibly via NPC transporters, and be trapped by sphingosine kinases (SKs) which phosphorylate it to S1P and/or by CerSs to generate ceramide outside the lysosome (84, 85). This reincorporation of sphingosine into sphingolipid metabolism is termed the salvage (or recycling) pathway.

In addition to the above basic metabolic pathway of synthesis and lysosomal degradation, which incidentally were the first to be appreciated historically, sphingolipids can undergo one of many regulated catabolic processes. Most notably, ceramides in various compartments (plasma membrane, Golgi, lysosomes, mitochondria) can be generated by the action of at least 5 distinct and compartment-specific sphingomyelinases (SMases) (86). Once formed, ceramides can be de-acylated by the action of one or more of 5 specific and distinct ceramidases (CDases) in these compartments (87, 88, 89), and this results in the formation of sphingosine which in turn can be either re-acylated to different ceramides in distinct compartments or phosphorylated by one of two SKs to form S1P. Cleavage of S1P by S1P lyase generates a fatty aldehyde and ethanolamine phosphate (90). This provides the only known metabolic exit out of the sphingolipid pathway.

As mentioned, the vast majority of sphingolipids are formed from the condensation of serine with palmitoyl-CoA; however, SPT is comprised of combinations of multiple subunits (SPTLC1 and SPTLC2 or SPTLC3) plus smaller subunits (ssSPTa or ssSPTb) and other interacting proteins that influence its substrate selectivity with respect to both the amino acid (serine, alanine or glycine) and fatty-acyl-CoA co-substrates and, therefore, the types of sphingoid bases that are made (21).

When alanine or glycine are used instead of serine, non-canonical 1-deoxy-,3-ketosphingoid bases are formed that undergo the other early steps of sphingolipid biosynthesis de novo (reduction and N-acylation), then follow different paths. N-acyl-1-deoxysphinganines (from alanine) are desaturated to N-acyl,1-deoxysphingosines with a Δ14Z double bond, whereas N-acyl-1-deoxymethylsphinganines (from glycine) are desaturated to N-acyl,1-deoxymethylsphingosines with a Δ4E double bond. Because of the absence of the C1-OH group, none of these 1-deoxy(dihydro)ceramides can be metabolized into more complex sphingolipids (11). These molecules are slowly catabolized through pathways involving, in part, cytochrome P450 (CYP)4F enzymes.

SPT is also the target of feedback mechanisms of regulation of de novo sphingolipid synthesis by the ORMDL proteins (Orms in yeast), which are negative modulators of SPT and respond to ceramide levels (91, 92, 93).

Although sphingolipids can be evaluated as a “self-contained” metabolic network, it is obvious that sphingolipid metabolism relies on and interacts with other metabolic pathways. For example, sphingolipid synthesis relies on serine, alanine, fatty acids, glucose and other sugars, choline, inositol, and other metabolites. These then serve to “connect” sphingolipid metabolism to many domains of intermediary metabolism. This was recently reviewed along with a discussion on functional consequences (94). Additionally, sphingolipid metabolism at the Golgi intersects with that of phosphatidyl inositol-4-phosphate (PtdIns(4)P) whereby PtdIns(4)P both controls and is controlled by SM synthesis in a feedback regulatory circuit that depends on the activity of CERT (which in turn responds to PtdIns(4)P), Protein Kinase D, PtdIns-4-kinase IIIβ (which locally increases PtdIns(4)P) and oxysterol-binding protein 1 (OSBP1) (95). In addition, transport of PtdIns(4)P from the Golgi to the ER by OSBP1 not only decreases PtdIns(4)P) in the Golgi but it is coupled to transport of cholesterol from the ER to the TGN, indirectly linking regulation of SM and cholesterol levels in the Golgi via PtdIns(4)P. An additional regulatory node linking sphingolipid biosynthesis and cholesterol levels was also established at the level of CerS2 activity in the ER (96). In the study, acute reduction of cholesterol levels caused a specific increase in the synthesis of very long chain (VLC) sphingolipids, mediated by CerS2 activity, ultimately leading to increased levels of VLC SM at the plasma membrane. The opposite was also true as loading of cholesterol suppressed synthesis of VLC sphingolipids revealing a metabolic connection that inversely regulates the synthesis of VLC-SPLs and cellular levels of cholesterol.

## Functions of sphingolipids and Mechanisms of Action

It is now appreciated that sphingolipids participate in a multitude of cellular and organismal processes, and the reader is referred to reviews on this topic (97, 98, 99, 100). Here we present a very brief overview of some of the key functions that are being defined for bioactive sphingolipids and mention some of the functions for more complex sphingolipids. We also present the current basic understanding of the mechanisms of action of bioactive sphingolipids.

### Signaling by bioactive sphingolipids

The earliest studies on ceramides employed cell permeable ceramides, and the results showed that these ceramides induced differentiation of leukemia cells in vitro (101). Then it was shown that they induced apoptosis of multiple cell types (102). Over the years ceramides have been mostly associated with growth suppression and with additional roles in mediating/regulating senescence, and cell cycle arrest (97), but also more recently in cell migration and adhesion (103). The *gestalt* of the actions of ceramides has been captured by considering ceramide as a *tumor suppressor lipid* in cancer biology (104, 105, 106). Outside cancer biology, ceramide has been implicated in inflammation, hormone action, cell stress responses, cell migration, and in countering actions of insulin (97, 98, 107, 108, 109).

Contemporary studies on biologic functions of ceramide have focused more on the role of endogenous ceramides, which are probed by determining effects of overexpression, knock down, or inhibition of key enzymes of ceramide formation or metabolism. We now appreciate that endogenous ceramide levels in cells are induced by a plethora of stimuli, including TNF, other cytokines, DNA damaging agents, other chemotherapeutics, and some hormones, and other stress stimuli such as ischemia/reperfusion (97). These stimuli regulate one or more enzymes of ceramide metabolism that result in the augmentation of ceramide levels. Many of these enzymes such as aCDase and GCS are emerging as therapeutic targets, especially in cancer (110, 111).

We also now appreciate that “ceramide” constitutes a family of closely related molecules that differ in their molecular structure, mostly with variations in chain length of the alkyl/sphingoid backbone and the acyl chain, but also variation in double bonds, hydroxylation, and saturation of the ceramide structure. This has led to the "Many Ceramides" hypothesis (71) that posits that distinct pathways of ceramide formation result in the formation of distinct ceramides in distinct compartments with different functional consequences. This is being supported by multiple contemporary studies (94).

In vitro, ceramide has been shown to activate serine/threonine protein phosphatases (CAPPs for ceramide-activated phosphatases), which have also been implicated in many cellular functions of ceramides (112, 113). However, the detailed molecular and structural mechanisms of how ceramide regulates protein phosphatases remain to be defined. Other targets for the direct action of ceramide have been proposed including PKCζ and cathepsin D (86); however, their roles in mediating ceramide responses are not clear, and there is little insight on how they are regulated in vitro.

On the other hand, S1P has been implicated in anti-apoptotic functions and in regulating angiogenesis, inflammatory responses, immune responses, cell migration, and resistance to chemotherapy (105, 114, 115, 116, 117, 118). As such, S1P has been advanced as a *tumor promoter lipid* in cancer biology, but obviously its actions are also involved in non-cancer biologies such as endothelial function, inflammation, infections and immunity. S1P levels are induced by growth factors (such as PDGF and EGF), G-protein-coupled receptor agonists (119, 120, 121, 122, 123), and cytokines (e.g. TNF) following activation of sphingosine kinases, especially SK1, but also the less studied SK2. Once produced, S1P functions primarily by acting on one of 5 G protein coupled receptors (S1PR1-5) (124, 125, 126, 127) in an autocrine and/or paracrine manner, which require its transport outside the cell through one or more transporters that include SPNS2, ABCC1, ABCG2, and MFSD2B (the latter in erythrocytes). S1P may also act on intracellular targets such as HDAC and TRAF2 (128).

Lysosphingolipids such as sphingosylphosphorylcholine (SPC or lyso-SM) have also been associated with signaling and the receptors for SPC are at least partially shared with S1P (34, 129, 130), Although sphingosine was the first bioactive sphingolipid to be identified (131), its participation in regulated pathways of signaling has lagged significantly behind the much better studied ceramide and S1P. Nevertheless, and similar to ceramide, sphingosine can mediate cell cycle arrest and apoptosis of a variety of cell types although we still know little about regulation of its generation and the mechanism by which it exerts anti-proliferative cellular effects (97). Notably, the CDase ACER2 is emerging as a regulated CDase that can produce pro-apoptotic sphingosine (132, 133, 134, 135). Sphingosine has also been found to be a regulator of Fam20C, an atypical protein kinase associated with the phosphorylation of multiple secreted proteins (136).

The bioactive sphingolipid C1P can be generated through the action of CERK. C1P is one of the least studied novel bioactive sphingolipids. Addition of C1P to cells exerts anti-apoptotic effects and enhances tumor cell invasion (137, 138) and cell migration. Exogenous C1P is required in the μM range (137, 138, 139). On the other hand, additional studies defined regulation of CERK by cytokines, including TNF, resulting in the formation of intracellular C1P, which has been implicated in the direct activation of phospholipase A2 (140), suggesting an intracellular action for C1P.

The functions of glycosphingolipids have been referred to as formation of "glycosynapses" (141), which are essentially functional domains that sometimes involve segregation of the glycosphingolipid(s) into domains in the membrane thought to regulate discrete receptor-mediated signaling (eg, "rafts") (more on these in the following section) and/or their direct binding to receptor proteins or other glycans to modulate receptor functions, ion channels, cell adhesion and cell-cell communication that influences growth, differentiation, immune interactions/signaling and cognitive functions (142, 143, 144, 145, 146). Much remains to be learned about the direct targets for specific GSLs, and mass spectrometry is one of the tools that will be useful in those investigations (147).

### Biophysical effects of sphingolipids

Sphingolipids are also important for their contributions to the biophysical properties of membranes, which also sometimes is an element of their participation in cell signaling (ie "glycosynapse") (141).

The biophysical properties of ceramides depend on both the nature of the sphingoid base (such as stronger intermolecular interactions for phytoceramides due to the extra hydroxyl group) and the N-acyl- chains (due to both chain length and double bonds, which affect lateral separation and fluidity) (148, 149, 150, 151). Therefore, variation of either/both of these can have large impacts on cell function, as discussed in this review and a recent article on the membrane biophysics of autophagy (152).

SM is quantitatively the major sphingolipid in most cells and lipoproteins, and studies with cells in culture and erythrocytes have found that a majority of the SM is on the outer leaflet of the plasma membrane, and normal cell morphology is sensitive to the acyl chain structure of SM (153). SM has a major influence on many cell functions (154), including the behavior of membrane cholesterol (155), the formation of membrane microdomains ("rafts") (156) and cell signaling, as discussed in this review.

Glycosphingolipids affect membrane biophysics by many of the same mechanisms mentioned for ceramides and SM with respect to fluidity and membrane microdomains, plus additional features provided by the bulky, sometimes negatively charged carbohydrate headgroups (from sialic acids and/or sulfate) (157). Interactions between the carbohydrate moieties and specific proteins that bind them (and sometimes also involving "raft" formation) affect structure and signaling in the same cellular membrane (which are referred to as *cis*-interactions) (for example, see reference (145)) and/or with extracellular matrix proteins and proteins on the surface of other cells (*trans*-interactions) functions (143, 146).

## Roles in disease

It is now becoming clear that sphingolipids are critically involved in a large number of diseases and pathophysiologies. Again, here we present the outlines of these involvements with the primary purpose of alerting the reader to the extensive field of research on sphingolipids and diseases (97). Broadly, we could organize this subject into four major categories.

### Inherited disorders of sphingolipid metabolism

The first group of diseases to emerge as involving sphingolipids were the lysosomal storage diseases where early studies on Niemann-Pick disease led to the identification of abnormal lysosomal storage of SM, subsequently traced to inherited defects in aSMase. This was followed by discovery of Gaucher’s disease, Krabbe’s disease, Tay-Sachs, Farber’s, Fabry’s, and others (158, 159). These all share the inherited loss of activity of an enzyme (or accessory protein) of lysosomal sphingolipid breakdown.

With the molecular identification of genes encoding many additional enzymes of sphingolipid metabolism, it is now appreciated that several inherited, non-lysosomal disorders of sphingolipid metabolism exist and involve alteration of SPT, individual CerSs, fatty acid alpha hydroxylase, alkaline CDases, S1P lyase, CERT1, Des1/DEGS1, and others (159), including congenital disorders of glycosylation (160). Genetic mutations of these sphingolipid genes can lead either to loss (so far more common) or activation of the protein (as in the case of CERT1 and the CerTra syndrome and SPT and Amyotrophic lateral sclerosis). Most of these are primarily neuronal disorders, except for S1P lyase deficiency which has primary systemic manifestations (kidney disorders) in addition to neuronal ones.

### Diseases where sphingolipids play pathogenetic roles

Appreciation of roles of sphingolipids in specific disease has been driven by two lines of independent research. The first is based on cell studies where a large body of literature now implicates/suggests roles for bioactive sphingolipids in various pathophysiologies, including inflammation, immunity, growth regulation, growth factor signaling (including insulin), response to cytotoxic injury (e.g. chemotherapeutics), cell migration and cell-to-cell attachments, stress responses, and many others. This line of investigation then progressed using in vivo models (mostly mouse but also including drosophila, *C. elegans*, and yeast). These now implicate sphingolipids in cancer pathogenesis and therapy, neurodegenerative disorders (e.g. Alzheimer’s disease and Parkinson’s disease), diabetes and metabolic syndrome, atherosclerosis and other cardiovascular diseases, multiple sclerosis, colitis, and many others (97).

A second line of investigation is a sphingolipid-agnostic approach whereby investigations into specific diseases led to identification of involvement of sphingolipids. This is well illustrated by Parkinson’s disease where it emerged that mutations in *GBA1*, in heterozygotes, constitute the largest group of inherited causes/predisposing factors for the disease (161). Another example is the identification of variants in SPT subunits that are associated with/cause ALS (162) and hereditary spastic paraplegia (163). Gangliosides, especially GM1, have been implicated in the pathogenesis of Guillain-Barré disease (164) and many other disorders (143).

Some glycosphingolipids are attachment sites for bacteria (165, 166) and viruses (including SARS-CoV-2) (167), and many disease-causing organisms produce toxins that target sphingolipid metabolism and sphingolipids, such as cholera and botulinum toxins (for gangliosides) (168), the fumonisin mycotoxins (for ceramide synthases) (169), and the sphingomyelinase D produced by some spiders, ticks, bacteria and fungi, which produce the novel metabolite ceramide 1,3-cyclic-phosphate (49).

### Sphingolipids as biomarkers

In the past decade, multiple omic studies have identified consistent changes in sphingolipids in specific diseases, most notably cancer, cardiovascular diseases, diabetes, metabolic syndrome, and Alzheimer’s. These have mostly focused on serum levels of ceramides and/or sphingomyelin, although some studies have identified changes in sphingolipids in cerebrospinal fluid in neurologic disorders. Notably, the Mayo Clinic has recently adopted measurement of plasma ceramides as an independent predictor of cardiovascular diseases and outcome in acute coronary syndromes (170, 171). Changes in specific glycosphingolipids have long been thought to play important roles in carcinogenesis and to serve as biomarkers of specific cancers (172) as well as cross-reacting antigens in some immune disorders (e.g. Guillan-Barré disease and celiac disease) (173).

### Sphingolipids in therapeutics

The increasing understanding of the roles of specific sphingolipids and specific enzymes in disease pathogenesis has opened doors towards novel therapeutics based on targeting sphingolipids and their pathways. The most advanced include the development of sphingosine analogs for the treatment of multiple sclerosis (in the clinic) (174), antagonists of S1P receptors in colitis (in the clinic) (175), enzyme replacement therapy for specific inherited disorders (e.g. Niemann-Pick disease) (176), and substrate reduction therapies in some of these disorders (most advanced for Gaucher’s) (177). Ongoing experimental therapeutics are focusing on delivering nanoliposomal ceramides and targeting key enzymes (e.g., SK1, Acid CDase, DegS1) in cancer (178).

## Complexities in the study of sphingolipids

### Analytical challenges

Lipidomics analysis is especially challenging for the sphingolipidome (179) because it is comprised of compounds with a wide range of functional moieties--i.e., lipid backbones with diversity in both the sphingoid base and fatty acyls and in the headgroups that include both phosphate esters and glycans, as discussed in the section “Structures of sphingolipids”. Therefore, sphingolipidomics studies only partially fulfill the formal definition of lipidomics as “the full characterization of lipid molecular species and of their biological roles with respect to expression of proteins involved in lipid metabolism and function, including gene regulation” (180). This fact of life is not problematic as long as the investigator matches the goals to the methods that are available, interprets the data rigorously, and informs readers about the scope of the study.

Whenever contemplating a lipidomic data set, one should think critically about how well the procedures have satisfied the recommendations for good practice in mass spectrometry (MS)-based lipidomics (181) as they apply to sphingolipids. One should start by asking:

1. Was the pre-extraction handling of the samples appropriate (prevented changes in labile subspecies during/after collection and during storage)?
2. How successful was the extraction of all the sphingolipid subclasses being analyzed? This refers to the wide range in amounts (over many orders of magnitude) and biophysical properties of sphingolipids (from ceramides and 1-deoxyceramides being some of the most hydrophobic lipids known to some lysosphingolipids and complex glycosphingolipids being compounds as or more soluble in water than in organic solvents.
3. Have all subcategories of sphingolipids been detected by the methods used (and associated questions regarding whether the amounts can be quantified from the ion abundances compared to appropriate standards)?
4. What artifacts might be created by the methodology (such as ionization suppression, in-source decomposition of labile compounds, etc.)?
5. What was the basis for the structural assignments, considering the many isomers and isobaric species to resolve (*eg*, double bond isomers, isomeric carbohydrates such as Glc and Gal, compositional and linkage isomers, etc.)? This issue becomes even more pronounced when these lipids undergo further metabolism into complex sphingolipids, and orthogonal techniques, such as liquid chromatography (LC) or derivatization, are essential for an accurate resolution and annotation of these structures.

Unfortunately, many publications do not provide sufficient information for readers to know the answers to these questions, and they might not be known by authors of the papers if they received the data from a collaborator or analytical service. One solution that has been proposed (182) is for lipidomics data to be accompanied by a checklist in common language that provides key information about how the lipidomic experiments were conducted and the data analyzed. Likewise, authors of lipidomics publications should provide comments about any aspect of the analyses that might limit the interpretation of the data.

The lipidomic methods that are currently available for analysis and quantitation of sphingolipids are too numerous to list and are changing rapidly, so only a brief overview is presented here. A major distinction between the methods is whether they are "untargeted" or "targeted". Both have advantages and disadvantages that have been discussed (183, 184).

In general, an untargeted lipidomic analysis (185) uses a single lipid extraction protocol followed by analysis of all the ionizable compounds by high resolution tandem mass spectrometry. The analysis is performed with or without use of an orthogonal separation step, such as liquid chromatography or ion mobility MS, which can also provide information about lipid backbone isomers in some instances (186). Lipid identification is obtained using the elution behavior and MS features (including fragmentation analysis for many methods) based on the match to one or more databases (many of which support both untargeted and targeted lipidomics analysis) (187). Untargeted analyses that assign structures using an identification algorithm sometimes suggest the presence of compounds that have never been seen previously in the biological system under study (and are likely isomers or isobars of other known compounds). Of course, unexpected findings might lead to an important discovery, but follow-up analyses are required before drawing that conclusion. In this context, results obtained using MS-based imaging techniques, such as Matrix-Assisted Laser Desorption/Ionization (MALDI) imaging, should be carefully analyzed and confirmed. In fact, while this technology provides valuable spatial information of sphingolipids in tissues and even in cells, MALDI data correspond to mass/ion (*m/z*) values. Therefore, the assignment of *m/z* values to specific sphingolipids should be carefully validated. In fact, the formation of artificial ions caused by laser-induced fragmentation (particularly when measuring low abundance sphingolipids such as ceramides, S1P and C1P) and/or the overlap with distinct isobaric molecules can cause mis-assignment of the signal (188). In the case of quantitative lipidomics, data should be looked at carefully to assure that factors that influence quantitation have been dealt with appropriately, including the use of adequate types and amounts of internal standards (189).

Most targeted analyses (see reference (57), for an example) use combinations of liquid chromatography and mass spectrometry to identify and accurately quantify a focused subset of sphingolipids (usually sphingomyelins, simple glycosphingolipids and the bioactive metabolites ceramides sphingoid bases and their phosphates). Conditions are optimized for 1) extraction of the specific compounds of interest; 2) chromatographic separation of compounds that might interfere with analysis due to ionization suppression or isomers with overlapping *m/z* (sometimes using more than one type of column and solvents); and 3) mass spectrometry protocols such as multiple reaction monitoring for quantitation of the analytes of interest relative to multiple internal standards. For example, with care in selection of the internal standards (189), it has been possible to estimate the average number of molecules per cell for multiple subspecies of sphingolipids using targeted LC-MS/MS (190).

Analysis of complex glycosphingolipids is one of the greatest challenges of sphingolipidomic methods (191). Historically, definitive identification of the glycans of glycosphingolipids has been determined by release of the aglycone (either lipid or protein to which the carbohydrates are attached) by chemical or enzymatic cleavage (192), followed by derivatization and analysis by a high-resolution MS method then comparison of the data with libraries of the types of glycans found in glycoproteins and glycolipids (193). This is being replaced by methods that are able to characterize both the lipid backbone and glycan headgroups (194), including novel ion activation methodologies (195). The use of ion trapping for MS/MS and MS^n^ analyses (ie, analysis of sequentially fragmented ions from the original glycolipid ion) helps identify the linkage position and other important information about each monosaccharide in the oligosaccharides (196). Another approach is to use a separation method that has been long-known to identify particular compounds, such as thin-layer chromatography (TLC) in combination with MS (197) (which has to be done with care because double bonds can undergo oxidation on TLC, although this can also be used to determine their localization (198)). Since histologic localization is often particularly important for analyses of glycosphingolipids in brain and other organs, methods that focus on small regions of tissue slices, such as microscale liquid extraction for surface analysis prior to mass spectrometry (199) and direct MS imaging (200) are powerful tools. Under special circumstances, even subcellular localization of specific sphingolipids can be achieved using secondary ionization MS (SIMS) (201). When doubts remain about glycan identifications, other methods can be useful, such as lectin or antibody arrays that trap specific glycan motifs (202, 203, 204).

Since lipidomics data are often collected by a variety of platforms, comparison of the results between studies of clinical samples can be difficult. This has been addressed in an interlaboratory study involving 31 diverse laboratories using different lipidomics workflows to analyze NIST SRM 1950 (National Institute of Standards and Technology Standard Reference Material 1950–Metabolites in Frozen Human Plasma) (205). The study found a substantial number of lipids for which the median of laboratory means (MEDM) had a sample coefficient of dispersion (COD, analogous to the sample coefficient of variation) > 40% and discussed possible reasons for the variation. A follow-up community-initiated position paper (206) addressed issues that should be considered in establishment of guidelines for clinical studies, such as reproducibility, accuracy and precision of lipid quantitation, study design, sample handling, and data sharing. An analysis of ceramides in another community effort (34 laboratories from 19 countries) (207) found that use of authentic labelled standards dramatically reduced data variability, and recalibration with a shared reference material decreased interlaboratory variability even further. Thus, although more optimization and validation studies are needed, substantial progress is being made toward identification of the factors that strengthen data for clinical and large cohort studies (208).

**Fig. 8:**
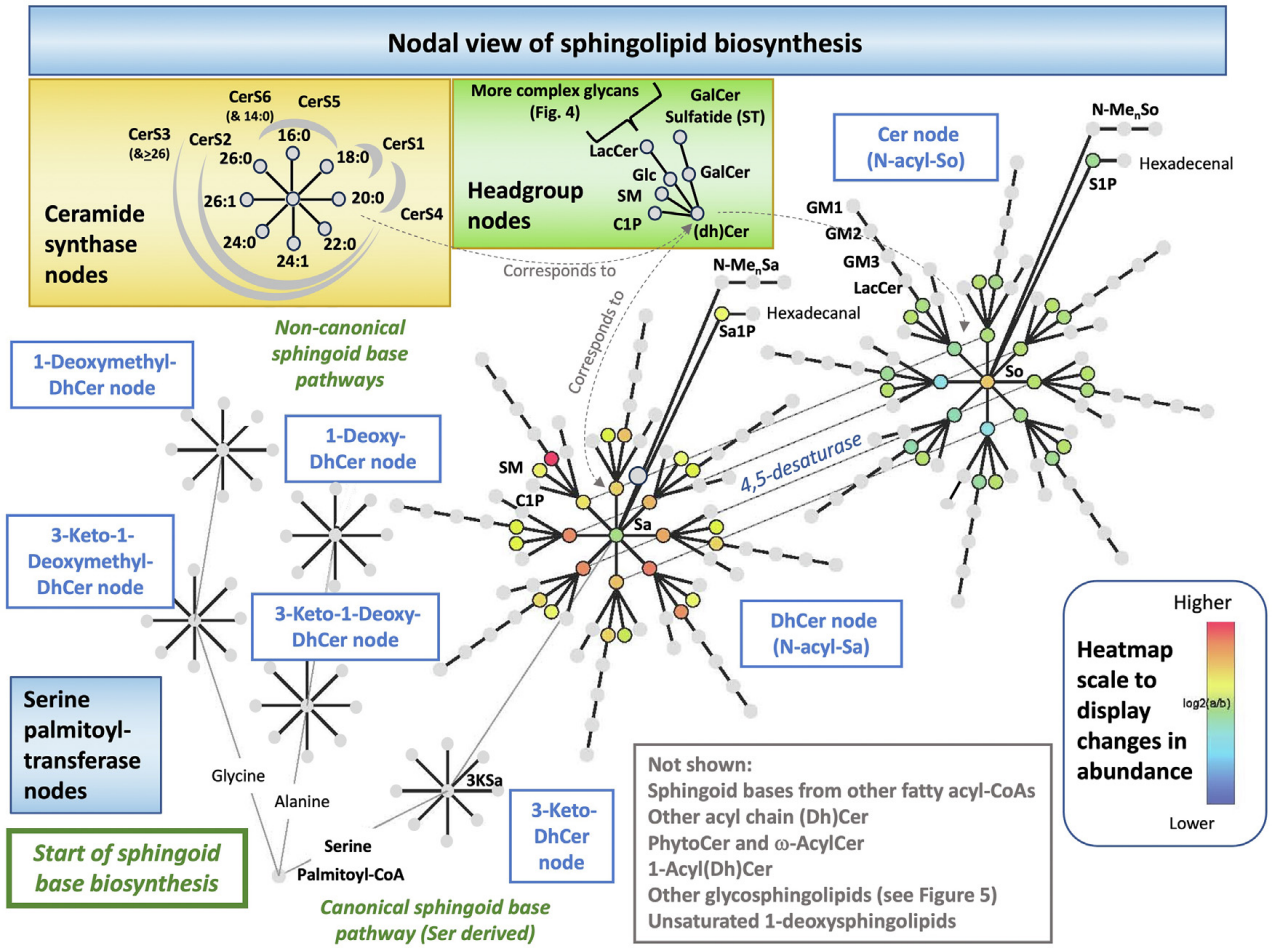
A pathway map for individual molecular subspecies of sphingolipids beginning from production of the sphingoid base backbone through subsequent metabolic steps, emphasizing key branchpoints (nodes). As in Figure 6, this diagram depicts sphingolipid metabolism but tracks the fate of every metabolite through each step and branchpoint. It begins in the lower left with condensation of serine and palmitoyl-CoA (noting the possibility of alternative precursors alanine, glycine and other fatty acyl-CoAs) to form 3-ketosphinganine (3KSa) (analogous processes shown for the keto intermediates of 1-deoxy- and 1-deoxymethyl-sphingoid bases, and analogous branches could be drawn for additional chain length sphingoid bases from other fatty acyl-CoAs). The 3-keto-intermediates can undergo reduction (for example, 3KSa to sphinganine, Sa) or N-acylation by ceramide synthases (CerS), which define a key branchpoint (see beige insert for the CerS nodes) that results in multiple chain-length 3-keto-dihydroceramides, 3-ketoDhCer (and 3-keto-1-deoxy-DhCer, etc.) depending on the substrate selectivities of the CerS and availability of the precursor fatty acyl-CoA's. For this figure, the CerS nodes depict the major fatty acyls of most mammalian tissues (16–26 carbon atoms) which are utilized by CerS1-6 (the gray arcs in the insert). Sa (and 1-deoxySa, etc.) are likewise N-acylated to DhCer (1-deoxy-DhCer, etc.) or phosphorylated to sphinganine 1-phosphate (Sa1P) (and possibly cleaved to hexadecanal) or N-methylated once or multiple times (N-Me-Sa: N-Mono-, Di-, and Tri-Methyl-Sa). DhCer can be metabolized to more complex sphingolipids (see insert for Headgroup nodes) or desaturated to Cer and also undergo headgroup addition; or Cer can be hydrolyzed to sphingosine (So), phosphorylated to S1P (and possibly cleaved to hexadecenal) or N-methylated (N-Me-So: N-Mono-, Di-, and Tri-Methyl-So). The initial headgroup options for (Dh)Cer are (see Headgroup nodes): Cer 1-phosphate (C1P), sphingomyelin (SM), Cer phosphoethanolamine (not shown), glucosylCer (GlcCer) and galactosylCer (GalCer); both GlcCer and GalCer can be further metabolized to more complex glycosphingolipids as shown by a few examples in this diagram (eg, lactosylCer: LacCer, and gangliosides GM3, GM2 and GM1 from GlcCer and a sulfatide, Gal-sulfatide) (for more downstream metabolites see Fig. 5). Note that the nodes for N-acyl-1-deoxy- sphingolipids do not display headgroup steps because they lack the 1-hydroxyl-group. When this scheme is used to depict lipidomics data (these data are from reference (210)), fold differences can be visualized for each analyzed compound using a heatmap with a color scale that represents the fold change. This particular data set compares the relative change in sphingolipids in MCF7 breast cancer cells treated with 10 μM Fenretinide (a desaturase inhibitor) for 24 h versus the control cells minus the drug. More information can be found in references (209, 210).

When a mass spectrometrist is pondering the meaning of changes in specific molecules in a lipidomic dataset, it might be helpful to visualize exactly where they are in the pathway. Figure 8 shows one format that has been proposed (209, 210) to depict the same pathway that is in Figure 6 but showing every molecule that is produced rather than generic subcategories such as "ceramides" or "sphingomyelins". Beginning in the lower left corner, it shows not only production of 3-ketosphinganine from Ser by SPT, but also the 3-keto-1-deoxy-sphingoid bases from alanine and glycine. Since these 3-keto-intermediates can have two fates, acylation by Cer synthases or first reduction then acylation (the major route in most circumstances), both are depicted as hubs with arms representing the different N-acyl-chain length variants. This type of depiction can be continued (theoretically) for every step of the pathway such as desaturation of dihydroceramides to ceramides (or alternatively, hydroxylation of dihydroceramides to phytoceramides, not shown) and addition of headgroups to produce more complex sphingolipids. The pathway diagram in this format can be constructed to include any combination of metabolites that have been detected in the lipidomics data set, such as additional N-acyl-chain ceramides, other chain length sphingoid base backbones, etc.

For those who are trying to track a specific molecular subspecies or cluster of metabolites found in a lipidomics study, this format might help clarify which perturbations alter the distribution of the subspecies; for examples, depletion of serine versus alanine and/or glycine to elevate 1-deoxy-sphingolipids; altered expression of the CerS isozymes to affect particular subspecies of DhCer, Cer and downstream complex sphingolipids; inhibition of DhCer desaturation to elevate dihydrosphingolipids, etc. Indeed, the heatmap depiction of Figure 8 (from (210)) shows results from the cells that were incubated with an inhibitor of DhCer desaturase and one immediately notices that all of the analyzed DhCer backbone sphingolipids are elevated and the Cer backbone sphingolipids decreased (c.f., the DhCer node vs. the Cer node). Other useful ways to visualize this pathway can be found on the LIPID MAPS online pathways (https://www.lipidmaps.org/resources/pathways) and other network maps (211).

### Functional and metabolic challenges

The involvement of sphingolipids in cellular functions as biomolecules whose levels are regulated can be thought of in terms of signaling modules whereby any one enzyme may act as a transducer of incoming signals (extra or intracellular), and upon activation or inhibition results in changes in the levels of its substrate and/or product. As such, one would hope to approach the study of specific enzymes and their products as isolated signaling modules, akin to the cAMP pathway. However, the study of sphingolipid-mediated pathways is characterized by several layers of complexity (86). This is because these are not isolated pathways with enzymes specifically dedicated to signaling and isolated from the rest of the sphingolipid metabolic pathways (Fig. 5, Fig. 6). Thus, the study of bioactive sphingolipids encounters several areas of complexity that we summarize here, but that have been discussed elsewhere (86).

#### Interconnected metabolism

All enzymes of sphingolipid metabolism are “connected” through the flux of the various sphingolipid metabolites. At a first level of approximation, sphingolipid metabolism has one primary entry point, the action of SPT (but also secondary entry of VLC fatty acids, glycans, phosphates, choline, and others) (94), and one major exit point through the action of S1P lyase (and cytochrome P450(CYP)4F enzymes for non-canonical sphingoid bases). Within this system, any particular sphingolipid can journey through the myriad paths of the sphingolipid metabolic network since the only “irreversible” reaction pathways are those of SPT and S1P lyase. Every other reaction connecting two sphingolipids in one direction has a counter pathway that operates in the reverse direction (e.g. SMSs and SMases, CerSs, and CDases).

This interconnectivity creates a “*Metabolic Ripple Effect*”. For example, activation of a SMase will generate ceramide, but if this fluxes rapidly through the action of CDase, then sphingosine may accumulate (or even S1P by the subsequent action of SK). Ceramide may also flux to C1P, GlcCer, SM, and acylceramide, thus illustrating the almost elusive nature of pinning the “real culprit” molecule. Therefore, one cannot conclude from results on changes in one enzyme that the action is due to changes in its product (see Fig. 2). This will require further dissection (see section “Approaches to unravel sphingolipid metabolism and function”).

#### Complex metabolism

The metabolism of bioactive sphingolipids involves the action of at least 40 enzymes that are products of 40 distinct genes, with obviously more forms that depend on post transcriptional changes and the formation of different complexes. Thus, manipulating one specific gene (as occurs in classical molecular biology approaches) may result in any of a number of possibilities of changes in sphingolipid metabolism. For example, down regulating or inhibiting SK1 may result in accumulation of not only sphingosine but also ceramide (and perhaps any of its metabolites). This is not as random as a “Whac-A-Mole” game because the changes in the sphingolipid network can theoretically and at times practically be predicted based on the activity of the enzymes in the network (212).

Nevertheless, the metabolism of sphingolipids can be simplified by focusing on the predominant pathways: de novo synthesis of ceramide and downstream sphingolipids, hydrolytic pathways for complex sphingolipids (especially SM) into ceramide and sphingosine, recycling/salvage of sphingosine into the sphingolipid network, and exit through the action of SKs and S1P lyase (Fig. 7).

#### Subcellular location

A major cause for the inflation in the number of enzymes of sphingolipid metabolism comes from the multiplicity of enzymes that share the same activity (eg 5 CDases, 5 SMases, 6 CerSs, etc.). These individual enzymes are products of distinct genes, and we are not even considering here splice variants or any post translational modifications. Importantly, these enzymes appear to reside in or favor distinct sub cellular compartments (e.g. SMases, CDases, SKs) (Fig. 7). There are significant implications to this phenomenon that arise from the hydrophobic nature of sphingolipids. These molecules will tend to reside in the compartments of their formation/generation unless they are trafficked (vesicularly, or by specific transport proteins) to additional compartments. The corollary to this compartmentalization of metabolism is that the functional consequences to changes in specific bioactive sphingolipids will be dictated to a large extent by what compartment they reside in. Thus, *compartment-specific metabolism* leads to *compartment-specific function.*

#### Multiple bioactive species

An important feature of bioactive sphingolipids as detailed in the section “Structures of sphingolipids” is the sheer number of their structural variants. We may on first study lump sphingolipids into specific subclasses: sphingomyelins, glycosphingolipids, ceramides, sphingoid bases, sphingoid base phosphates, and ceramide phosphates. However, and thus far mostly for ceramides and sphingoid bases, we are learning that their structural variations can portend distinct functions. It should be noted that these structural variations are introduced by specific genes. For example, the variation in the length of the acyl group of ceramide is due to the selective substrate specificity of each of the 6 CerSs and not due to random insertion of different acyl chains by any one CerS. Other variations include specific desaturation and/or hydroxylations of the acyl chain and the sphingoid backbone and variations introduced by SPT acting on alanine or glycine instead of serine. Since as far as we know these groups of variations are mostly not mutually exclusive, then the possible number of ceramide variants can reach the 300 range (Fig. 4). The sphingoid bases are significantly less complicated than the ceramides, yet with the inclusion of the 1-deoxy-sphingoid bases and their modifications and other double bonds, and so on, the numbers may add up to more than a dozen.

#### Very low levels

To further complicate their study, many sphingolipids (sphingoid bases, their phosphates, individual ceramide species) exist in very low levels of less than 0.1% of total cellular lipids (and for some, much less). The low abundance sphingolipids could be present for a variety of reasons: some are made in low amounts due to the potency of their biological function (for example, S1P as a signaling lipid, or highly specialized glycosphingolipids); many are produced in small amounts by biosynthetic enzymes that have reasonably high, but not absolute, substrate specificities (for example, d17-and d19-odd-chain-length sphingoid bases made by utilization of pentadecanoyl- or heptadecanoyl-CoA's by serine palmitoyltransferase); and, some are compounds that have been taken up and reutilized from exogenous sources, such as sphingolipids in food or produced by intestinal microflora. These considerations necessitate careful analytical approaches which for the most part have relied on MS.

Despite their low abundance, sphingolipids from exogenous sources are noteworthy because they have been found to be taken up from the diet and impact disease. For example, studies with experimental animals have demonstrated that diets with multiple subcategories of sphingolipids (sphingomyelins and glycosphingolipids) (213, 214) reduce chemically induced or hereditary colon cancer, and the levels of sphingolipids used in these studies appear to be in the same range as sphingolipids in human diets (215, 216). Moreover, sphingadienes such as those found in soy (with 4E,8Z-double bonds) appear to be particularly effective in models for colon cancer (217) and neuroblastoma (218). While this is somewhat surprising because of the existence of an intestinal efflux mechanism (P-glycoprotein) for sphingoid bases other than sphingosine (26), the dienes have been found to undergo intestinal uptake (25, 219, 220), nevertheless. Intestinal microflora are also important producers of sphingolipids that can be taken up and affect the host, as exemplified by findings with the potent immunomodulator α-GalCer, which is produced by the *Bacteroides fragilis*, a member of the human gut microbiome (54, 221). These are just a few examples of instances where exogenous sphingolipids have been found to affect physiologic functions, and lipidomics analyses will need to take these into account.

#### Physico-chemical properties

The hydrophobic nature of bioactive sphingolipids and the charged and/or bulky headgroups of phosphorylated and complex sphingolipids impede the application of usual and simple cell biology and pharmacology approaches (222, 223). Many of these molecules are not easy to dissolve in media-compatible forms, and they are not assured translocation across the plasma membrane or that they reach the intended sub cellular compartment. Because of the hydrophobic characteristics that limit the diffusion of sphingolipid within the cells, sphingolipid concentrations should be considered in the context of the lipid-rich environment in which they reside (for further details see Box 2).

Box 2 **The concept of mole percent when referring to lipid concentrations.**

Hydrophobic lipids will partition into lipid rich domains (membranes in cells or liposomes or micelles in vitro) instead of remaining in solution. Therefore, their biologically effective concentration is better calculated in moles percent of total lipids rather than molarity. Mole percent is the mole amount of a certain lipid of interest divided by the total lipid moles x 100.

## Approaches to Unravel Sphingolipid Metabolism and Function

In this main section, we address the issues that arise when an experimental biologist confronts results that suggest sphingolipids and/or sphingolipid enzymes are important for their studies. As discussed at the outset, non-lipidologists encounter sphingolipids usually following an “omic” screen which can take the form of lipidomic/metabolomic studies and/or genomic/proteomic studies (which include studies on discovering new disease-causing or associated genes) (Fig. 2) and/or studies involving inhibitors or CRISPR screens for targets. Here we address the key questions that arise from such “inputs,” especially as first encounters with the field of sphingolipids.

A. How to assess changes and perturbations in sphingolipid levels and advance these approaches?
B. How to evaluate and probe changes in enzymes of sphingolipid metabolism and link them to changes in levels of bioactive sphingolipids?
C. How to establish the contributions of these changes to the biology under study, ie, how to determine the functional significance of changes in the sphingolipidome (enzymes and/or lipids)?

But before we get into this first level of investigation, we posit that for advanced resolution into the mechanisms and functions of specific sphingolipids and for more optimal resolution of the cellular operation of these pathways, one must address the following additional but important issues which are the ultimate goals of most sphingolipidologists. These are not addressed in this review.

1. How does the process on hand (e.g., DNA damage response) modulate bioactive sphingolipids and their enzymes? In essence, this is a question about ‘*upstream mechanisms*’ that are involved in regulating specific enzymes in the sphingolipid pathway.
2. How do each of the implicated sphingolipids function to mediate specific responses or components of those responses? This is a question about ‘*downstream mechanisms*’ of direct and indirect actions of the implicated bioactive sphingolipid.
3. For even more in depth understanding, given the compartmentalization of sphingolipid metabolism, an important set of questions emerges as to *where in the cell do these sphingolipid-mediated pathways operate*?

### Assessing changes and perturbations of sphingolipid metabolism and advancing these approaches. Mass versus flux

There are two major reasons to expend effort on defining changes in levels or metabolism of sphingolipids, especially the bioactive sphingolipids. First, changes in sphingolipid levels provide evidence that the biology under study affects sphingolipid pathways and therefore may involve the action of some bioactive sphingolipids in the process. Second, this paves the way towards defining the functional impact of modulating sphingolipid metabolism since, functionally and mechanistically, it is the lipids whose levels are changing that in effect mediate the functions of those enzymes and pathways. We have advanced this as the *Lipid-Centric Approach* where we define what sphingolipids are changing and especially which ones function as a mediator of specific responses in the biologic process under investigation (71, 103, 224).

Perturbations of sphingolipid metabolism can result in: 1) changes of mass levels of specific lipids and/or 2) changes in the flux through the interconnected reactions. Changes in mass levels are the results of changes in the flux through the pathway; however, changes in the flux through components of the pathway may not produce mass changes at least in some of the direct metabolites.

#### Mass changes

Currently, *mass changes* are being determined mostly by combining liquid chromatography and MS. As discussed in the section “Analytical challenges,” this provides the most comprehensive and quantitative approach to assess the various molecular species of the different classes of sphingolipids.

Additionally, measurements of relative mass levels can be obtained utilizing other approaches. Cellular sphingolipids can be labeled to a *dynamic steady state* with radioactive or isotopically labeled precursors. Steady state is reached once the labeled precursor, present in trace amounts, is taken up by the cells and incorporated into the target lipid biosynthetic pathway to form a pool of labeled target lipid in pseudo-equilibrium with the unlabeled endogenous pools of the same lipid. In this case, a difference between the amount of labeled target lipid in control vs. treatment is an indication of mass changes caused by the treatment. Some words of caution. 1) Labeled precursors should be added in trace amounts so as not to alter endogenous metabolism due to a mass effect of the precursor itself. 2) It is critical to maintain an excess of labeled precursor, not to inadvertently initiate chase of the labeled pools. 3) It should also be kept in mind that labeled precursors will more efficiently incorporate into metabolically active pools of sphingolipids potentially failing to capture long lived sub-pools.

Other tools/approaches allow the measurement of relative sphingolipid levels while also assessing their subcellular localization (this is mostly for ceramide, SM and glycosphingolipids).

i) Sphingolipid binding toxins can be employed to stain target sphingolipids and indirectly measure their levels. Among these are Ostreolysin A6 (225) and Nakanori (for SM/Cholesterol complexes) (226); Equinatoxin II and the E69A mutant of Ostreolysin A6 (for dispersed SM, and CPE for E69A) (227, 228, 229, 230); lysenin (for clustered SM) (231, 232), Shiga toxin B subunit (for Gb3) (233) and cholera toxin B subunit (for GM1) (234). There are well established protocols for these that have been repeatedly tested (234).
ii) Sphingolipid-recognizing antibodies are used for immunofluorescence, immunoEM, and flow cytometry. Among these are antibodies recognizing ceramide and the glycosphingolipids LacCer, Gb3, and GM3. For ceramide, given its low local abundance, the antibodies need to be carefully evaluated for specificity with positive and negative controls. In our hands, a very limited number of antibodies deliver a ceramide specific signal (235).
iii) A recently developed method applies purified bacterial SMase and CDase to fixed cells to measure levels of SM (by quantifying SM loss and/or ceramide formation) or ceramide (by measuring its conversion into sphingosine) in the outer leaflet of the plasma membrane, respectively (236). The main precaution is the need for using excess CDase to convert all the ceramide to sphingosine.
iv) In case of tissue samples, 3D-cell structures and single cells, MS-based imaging approaches are also available (237, 238). While most sphingolipids may be detected with this methodology, detection of sphingosine, S1P and C1P requires careful consideration, and these approaches are still under development.

#### Flux analysis to study de novo sphingolipid biosynthesis and degradative pathways

Investigating which of the reactions within the pathway are impacted by a specific experimental condition often requires an assessment of the changes in flux through the sphingolipid pathway or through nodes of the pathway (defined here as key steps or branches of sphingolipid metabolism that can be regulated by more than one specific enzyme). Flux analysis allows an evaluation of the rates of the different reactions through the network.

Different approaches are applied depending on whether the need is to measure biosynthetic versus catabolic reactions, and protocols can be further focused on specific enzymes/nodes in the sphingolipid pathway as discussed below. Details of approaches to analyze flux through specific enzymes are presented in (239).

##### Biosynthesis

The biosynthetic branches of the sphingolipid pathway can be probed via “pulse” labeling. There are several precursors that can be utilized depending on which node of the pathway is to be probed (Fig. 5).

Early precursors such as isotopically or radioactively labeled serine or fatty acids allow us to follow several consecutive reactions in the de novo pathway, starting with SPT. Likewise, the use of d17dhSph or d17Sph probes the reactions downstream of generation of sphingoid bases and allows the focus on the actions of ceramide synthases, desaturases, sphingosine kinases, and downstream metabolites. On the other hand, use of fluorescently labeled short-chain ceramide analogues (NBD- and BODIPY Ceramides) allows probing of more downstream (individual or branched) reactions, especially in the Golgi compartment (see Box 3 for specifics of approaches).

Box 3 **Tool box.**

Probing Sphingolipid Biosynthesis.

In an early study, ^14^C-serine was used to follow the synthesis of sphingoid bases and ceramide, and this work actually led to establishing the sequence of formation of dihydroceramide first, then desaturation to ceramide (as opposed to direct saturation of sphinganine) (240). More recently, an assay was developed using deuterium-labeled L-serine (D_2_-serine) followed by LC/MS/MS to follow incorporation of serine through SPT into 3-ketodihydrosphingosine and then into subsequent metabolites (241). This was particularly useful in detecting the very early metabolites in de novo synthesis of sphingolipids and in clarifying the role of the Tsc3 small subunit of SPT.

A variation of this approach has also been recently utilized to assess changes in overall de novo sphingolipid synthesis versus changes due to catabolic reactions (21, 242). Isotope-labeled D_3_-^15^N-serine was added as precursor; this molecule loses one deuterium in the course of the first reaction of de novo synthesis, giving rise to newly synthesized sphingolipids with a +3 Da (+3D) shift in mass. In this approach, after pulsing cells with isotope-labelled L-serine, total levels of newly synthesized (+3D, heavy) and steady state (light) (dihydro)sphingolipids were quantified as a function of the amount of total sphingoid bases (dihydrosphingosine/sphinganine and sphingosine) released after chemical acid breakdown of extracted lipids. Changes in +3D (heavy) versus endogenous (light) sphingoid bases provide therefore an indication of overall alterations of de novo synthesis versus catabolic metabolism.

Labeling cells with d17dhSph, a sphingoid base with an odd number of carbons rarely present endogenously in substantial amounts, allows the assessment of enzymatic rates through various sphingolipid enzymes. As soon as it is administered to the cells in trace amounts, d17dhSph is rapidly converted into d17dhCer by ceramide synthases (0–15 min). D17dhCer is then converted into d17Cer by the dihydroceramide desaturase (20–60 min), while d17Cer is further utilized by the sphingolipid metabolizing enzymes in the Golgi and converted into complex d17-sphingolipids (60–120 min). Using this approach, modulation of one or more steps of the pathway can be contemporaneously assessed and measured by the change in rate of conversion of each individual labelled substrate into the respective labeled sphingolipid product. As an example of using such an approach, the regulation of multiple nodes of the sphingolipid pathway by doxorubicin was demonstrated in MCF-7 cells (243). Moreover, in case of the CerS phase, the combination of pulse labeling with information on fatty acyl chains of the various d17dhceramide provided by MS may infer specific modulation of the different CerSs based on the distinct substrate (fatty acyl CoA) specificity of individual CerSs. It is to be noted that it is essential to probe each specific node in the de novo pathway focusing on the linear phase of the pulse labeling of that enzyme/node; otherwise, changes in kinetics may be masked by steady state levels. The appropriate phases for a typical cell (MCF7) are given above; however, these may need to be validated or defined when using other cell types. Please refer to Snider *et al.* (244) for an example.

Recently, functionalized sphingoid bases have also been used to assess sphingolipid metabolism. These sphingolipid analogs contain different functional groups (bi-functional photoactivatable and clickable -PAC- sphingolipids or tri-functional sphingolipids with addition of a caging moiety) that allow investigators to follow sphingolipid metabolism and localization and also to trap interacting proteins (245) (and see (246) for review). Chemical protection with use of a photocage at the amine group prevents these tri-functional precursors to be converted into (dh)ceramides as they are added to cells. Once in the cells, removal of the protecting group (*uncaging*) by shining light of specific wavelength (depending on the cage) onto cells frees the analogs which can now enter endogenous metabolic routes. Incubation of these analogues in cells for different times after uncaging allows analysis of their metabolism by MS. Furthermore, a terminal alkyne moiety on these analogs enables click chemistry to add other functional groups for analysis of metabolism by thin layer chromatography (TLC) and/or intracellular localization (ie linkage of a fluorophore allows the visualization of the metabolites on TLC and within the cell). Modification of the photocage with organelle-targeting moieties has also provided metabolic information specific to mitochondrial and lysosomal sphingolipid metabolism (247, 248). Finally, the presence of a diazirine on the sphingoid backbone allows photocrosslinking with interacting proteins which can be identified by proteomics analysis. Recently, the caSph was also developed where a photoactivatable conversion of straight *trans* to curved *cis* configuration of the sphingoid backbone is made possible by insertion of an azobenzene and initiated by light activation; the isomeric switch allows the real-time enhanced utilization of the *cis* probe by CerSs for Cer synthesis and down-stream metabolism (249). This probe has the advantage of being reversible (it can be switched on and off) and it does not release byproducts in the cell (ie cage moieties).

It should be noted that all functional sphingoid bases, with a 1-OH group, go through substantial metabolic elimination via conversion into S1P and subsequent breakdown by the S1P lyase; depending on its efficiency, this catabolic node may limit the overall accumulation of these probes in cells.

FRET as readout of sphingolipid enzymatic activities.

In one example for aSMase activity, the substrate SM carries the NBD and coumarin fluorophores at the headgroup and the fatty acyl chain, respectively; when SM is intact, excitation of the coumarin causes an energy transfer that in turn excites the NBD fluorophore with observed emission at 560nm. Following SM hydrolysis, the loss of the headgroup causes a decrease of NBD-fluorescence and a parallel gain of coumarin fluorescence (emission at 410nm) (250). A drawback for this FRET pair is the need for a two-photon excitation microscope; however, a promising recent advance introduced a novel FRET pair in the visible range, BODIPY and Fluorescein, which allowed measurement of aSMase activity *in cellulo* using flow cytometry (251). In another example for evaluating CerS activity, NBD-labeled 1-deoxydihydrosphingosine (spisulosine) was used as a substrate together with palmitic acid carrying either Nile Red or coumarin in the omega position (252). Since 1-deoxysphingolipids cannot be further metabolized into complex sphingolipids, they are particularly well suited as reporters for CerS activity.

##### Catabolism and Turnover

Catabolic sphingolipid nodes can be assessed by using a “chase” phase of labeling. Once steady state labeling is reached for the specific sphingolipid of interest, the chase phase is initiated with the removal of the labeled precursor from the medium. In this case, the rate of disappearance of the labeled target sphingolipids measures their turnover rate and is an indication of the activity of the specific catabolic enzyme.

Traditionally, flux analysis has been applied mostly to study sphingomyelinase activity following steady state labeling with tritiated choline. However, this is an underdeveloped area that necessitates enlargement of the scope to address other classes of sphingolipids and the development of more sophisticated protocols.

Given the fact that each metabolic reaction can be catalyzed by multiple isoforms of the same enzyme and/or different enzymes, these *in cellulo* approaches will not necessarily identify specific enzymes but may pin-point certain nodes of regulation. Further targeted analysis is needed to identify the specific enzymes/proteins being modulated in the experimental condition under investigation with strategies discussed below.

### Evaluating and probing modulation of sphingolipid-metabolizing enzymes

Evaluation of changes in sphingolipid levels or flux often leads to questions about which specific enzyme is involved. Alternatively, investigators may first encounter changes in specific enzymes of sphingolipid metabolism during the course of their studies. In either case, one needs to address important and specific questions as to 1) is a specific enzyme of sphingolipid metabolism regulated? 2) how does it affect sphingolipid metabolism and levels? These then lead to questions on 3) What is the role of the target enzyme in mediating or regulating the processes being studied (discussed in the section “Defining the contributions of these changes to the biology under study”)? 4) where in the cell does this occur? (not discussed here), and 5) what are the mechanisms by which the stimulus or perturbation under study modulate specific enzymes of sphingolipid metabolism? (not discussed here). We have previously articulated this as an *Enzyme-Centric Approach* to define what are key enzymes involved (71).

The regulation of a specific enzyme can be approached at both a cellular level to establish the existence of this regulation in cells and could be approached in cell-free systems to further establish the regulation of specific enzymes and extend the scope of the studies.

#### Cellular approaches

Different precursors and/or labeling approaches can be applied not only to study the global changes in the sphingolipid pathway as discussed in the section “Flux analysis to study de novo sphingolipid biosynthesis and degradative pathways,” but they can also be catered to assess specific enzymatic activities or discrete nodes.

For example, as discussed in the section “Flux analysis to study de novo sphingolipid biosynthesis and degradative pathways,” SPT activity can be monitored with acute labeling using serine or palmitate (or other amino acids or fatty acids) and CerSs can be followed by using d17dhSph (or d17Sph). In studies of this type, it is informative to determine the relationship between the amount of added probe and the endogenous unlabeled equivalent (ie, the specific activity or isotopic enrichment, etc.), whether that be serine (253), fatty acyl-CoAs (254) or other relevant substrates.

A powerful assay using cationic pyridinium C12-dihydroceramide was developed to evaluate *in cellulo* DES activity with high precision and, depending on the formation of pyridinium C12-ceramide versus pyridinium C12-hydroxyceramide, it can discriminate between the activity of DeS1 and 2, respectively (255, 243).

A short pulse (10–30 min) with d17-Sph (ideally added in the nM range) can be used to measure SK activity by MS (239, 256).

The use of fluorescently labeled short-chain ceramide analogs (NBD and BODIPY which concentrate in the Golgi apparatus (257)) allows the measurement of Golgi-localized activities such as SMS, GCS, and CERK (258, 259, 260, 261).

Assessment of the activity of the ceramide transfer protein CERT1 can be probed using BODIPY-ceramide. Adding BODIPY-ceramide at 4°C for a short duration (10–30 min) prior to incubation at 33–37°C, leads BODIPY-ceramide to localize in the ER and to be synchronously transported to the Golgi via CERT1 once the temperature is shifted to 33–37°C (262, 263, 264). Under these conditions, formation of BODIPY-SM is dominant over GlcCer and dependent on CERT1.

A “cold-loading” approach coupled to “lipid back-exchange” may be utilized for measuring SMS2 activity at the outer leaflet of the plasma membrane. In this case, intact cells are incubated at low temperature (4°C) with NBD-C6-Ceramide complexed to fatty acids free bovine serum albumin (BSA) in order to prevent endocytosis while allowing metabolism at the plasma membrane. The amount of NBD-C6-SM in the loading buffer and after the back-exchange is a measure of SMS2 activity (265, 266).

Alternative approaches have measured *in cellulo* enzymatic activities exploiting quenching of sphingolipid probes, where probes are either turned on or off by the targeted enzyme. A recent development in this direction is the use of Förster (fluorescence) resonance energy transfer (FRET) acceptor and donor probes which enable the measurement of both the decrease of the substrate and the formation of the product (Box 3). This technique has been utilized to measure aSMase activity with NBD/coumarin or BODIPY/fluorescein fluorophore pairs and CerS activity with NBD/Nile Red or NBD/coumarin pairs (250, 251, 252).

#### Cell free approaches

More classically, regulation of sphingolipid genes/proteins can be studied by assessing changes in their levels of expression by Real-Time PCR (mRNA) or by western blotting (proteins). In addition to targeted real-time PCR, the use of sphingolipid gene arrays has also proven valuable in revealing more global changes in response to specific treatment (243).

When using western blotting, great care should be given to validation of antibodies as the endogenous levels of many sphingolipid proteins are low. This should include evaluation of the antibody against overexpressed or purified protein as well as loss of signal with down regulation of the target. Subcellular fractionation may be needed to enrich the level of the protein of interest (this is the case for SMSs and ACERs). Also, in addition to western blotting, a recently published protocol has begun to apply MS to measure the levels of some SPL enzymes (267); however, this requires additional development.

Another tool to probe regulation of sphingolipid enzymes is measuring their enzymatic activities in vitro on lysates and/or subcellular fractions. Importantly, specific experimental conditions allow the discrimination between activities of different isoenzymes. This is the case of SK1 (inhibited by KCl) and SK2 (inhibited by CHAPS) which can be discriminated by different assays conditions (268, 269). DeS1 and 2 can be distinguished by utilizing the same substrate (pyridinium C12-dihydroceramide) and measuring different products by MS (pyridinium C12-ceramide vs. pyridinium C12-hydroxyceramide, respectively) (243). Also, the various CerSs can be assayed taking advantage of their different fatty acid specificity.

Other isoenzymes can be distinguished by their pH optimum and/or lipid activators such as acidic versus neutral pH for aSMase and neutral SMase (nSMase), respectively or dependence on phosphatidylserine for nSMase 2 and not nSMase 1. Also, different forms of aSMase (lysosomal vs. secreted) can be distinguished due to the Zinc dependence of the secretory form (270).

On the other hand, some sphingolipid enzymes, such as SMS 1 and 2 cannot be distinguished in vitro, and therefore the in vitro activity is the sum of the two isoenzymes. Defining the contribution of one versus the other may require suppressing the expression of one of the pair at a time.

It is important to keep in mind though that a drawback of measuring in vitro enzymatic activity is that potential regulation by cofactors, allosteric regulators, or inhibitors might be lost during the preparation of the sample. Furthermore, if the activity of an enzyme is modulated by a change in localization that leads to its activation or inhibition, an in vitro assay will likely fail to capture this level of regulation. For these considerations, assessing the activity of an enzyme directly in cells (*in cellulo*), as discussed in the previous section, is recommended whenever possible.

### Defining the contributions of these changes to the biology under study

Once a specific enzyme is demonstrated or suspected to be regulated by the process under study, it usually becomes important to link its activity to a specific sphingolipid profile and to define its functions and contributions to the process under study. Two general approaches are currently available: modulation of enzyme expression or function, and/or inhibitor studies.

With the identification of all/most sphingolipid genes, modulation of gene expression with silencing RNA or CRISPR/Cas9 technologies provides a powerful tool to link specific sphingolipid profiles or flux changes to enzymatic activities and eventually cellular processes. Caution is required, however, especially when results with siRNA-mediated knock down diverge from results obtained with highly specific inhibitors, as observed on more than one occasion with SK1 (271, 272). Thus, multiple siRNAs are needed as well as other complementary approaches (e.g. CRISPR/Cas9-mediated knock down or use of cells from knock out mice).

Importantly, several inhibitors targeting most major sphingolipid nodes have been developed. While the most specific ones currently available are indicated in Fig. 5, many other inhibitors have been utilized. In this regard, it is important to stress the importance of validating the effectiveness of the inhibitor against the intended target whenever possible and to utilize the minimal effective concentration to avoid off target effects. In this context, obtaining a biologic response at concentrations in significant excess of what is needed to inhibit the target in cells (often 10 times higher or more) actually argues for negating a role for that enzyme. Reference concentrations for most inhibitors have been previously reported (239, 258, 273, 274).

An often-used complementary approach to the modulation of specific enzymatic activity is directed at mimicking the observed sphingolipid changes (in the absence of direct enzyme manipulation) and assessing its biological consequences. In the case of ceramide and sphingosine, this approach may utilize addition of exogenous (cell permeable) analogues or bacterial sphingolipid enzymes such as SMase and CDase (mostly extracellularly). While this strategy has been valuable (particularly early on in sphingolipid research) to establish the bioactivity of these molecules, caution should be used. When adding sphingolipid analogues to cells, consideration should be given as to how the concentration used relates to the changes of endogenous sphingolipids, and using excessive concentrations of the target lipid significantly beyond their cellular concentrations can elicit off-target effects although surprisingly (at least in the case of ceramide), hardly any have been discovered after decades of use. Nonetheless, it is important to demonstrate the “specificity” of the added lipid by using closely related analogs and stereoisomers if possible. Furthermore, the use of these exogenous sphingolipids can/should be augmented by manipulating their metabolism. For example, biologic responses generated by adding exogenous sphingosine should be evaluated by adding specific inhibitors of its metabolism (e.g. PF543 to inhibit SK1 or Fumonisin B1 to inhibit CerSs). If one of the inhibitors blocks the action, the results then suggest a key role for the downstream metabolite (S1P of ceramide, respectively in this case). However, when using an inhibitor, the accumulation of upstream precursors that are likely to be bioactive compounds may affect important biochemical processes, thus, further complicating the interpretation of the results (169). In the ideal situation, exogenous addition of the presumed sphingolipid of importance for a biological process will restore function at realistic concentrations (for example, nM concentrations of S1P to study S1P-receptor’s activity since that corresponds to the KD of S1P binding to its receptors). Nonetheless, one also has to be mindful of the biophysical properties of the compounds and their physiological milieu when adding lipid analogs to cells. For instance, in the case of delivery of S1P to its receptors, this may require complexation with fatty acid-free BSA or high-density lipoprotein (HDL) to mimic the bound form of the lipid naturally occurring in serum.

Because of these considerations, it is advisable to utilize these pharmacologic approaches in conjunction with genetic tools and/or inhibitors targeting the enzymes of interest as discussed above.

As discussed in the section “Complexities in the study of sphingolipids,” the compartmentalized distribution of sphingolipid enzymes and the chemical nature of sphingolipids favor localized lipid changes. Therefore, addition of cell permeable analogues which may distribute in various compartments within the cell or accumulation of sphingolipid in the plasma membrane by treating with exogenously added bacterial sphingolipid enzymes may not necessarily reflect the changes invoked by the enzyme under study. Advancements to address this limitation and trigger lipid changes in a more localized fashion have seen: 1) the design of positively charged ceramide to study its functions in mitochondria (275, 276, 277); and 2) expression of sphingolipid enzymes (mostly bacterial but also CERT1) engineered to localize in specific subcellular compartments/organelles (103, 278, 279).

In summary, the field of dissecting sphingolipid changes and deciphering the functions of key enzymes and bioactive sphingolipids is experiencing tremendous technological advances (eg, advanced LC/MS/MS, flux analysis, bi- and trifunctional precursors, MS-based imaging, CRISPR, compartmentally targeted probes and enzymes, etc.) often supported by the emerging utilization of machine learning/artificial intelligence tools. These are allowing the progress of the field into tackling what we believe are key gaps such as defining compartment-specific functions, the interconnectedness of bioactive sphingolipids (and also consideration of points of integration with other cellular metabolic pathways) and advancing mechanisms of regulation of enzymes and mechanisms of function of bioactive sphingolipids.

## Perspectives

**Fig. 9:**
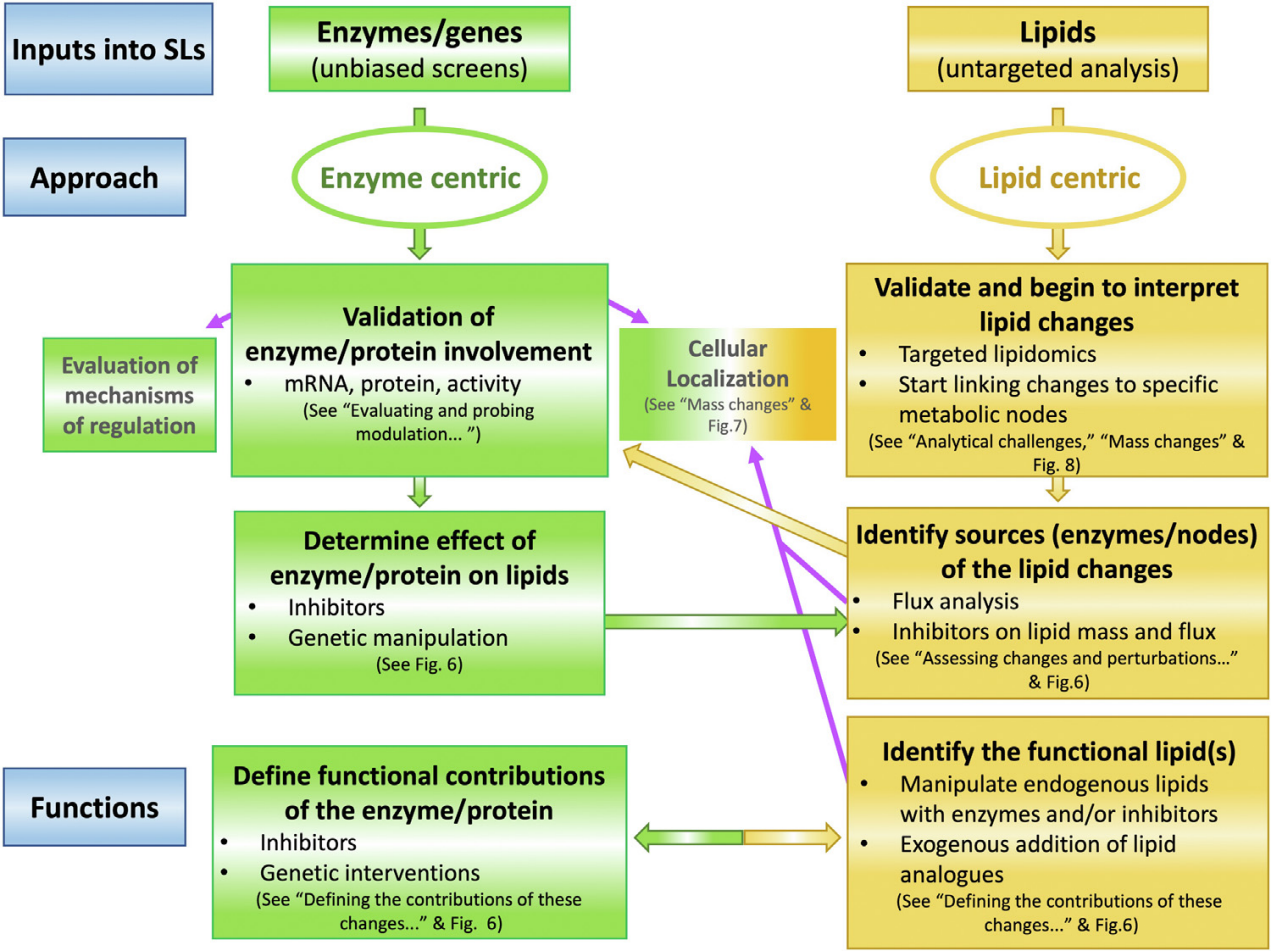
Workflow illustrating experimental paths that support rigorous interpretation of sphingolipid changes and advancement of these discoveries. A change in either sphingolipids (by untargeted metabolomics/lipidomics) or sphingolipid enzymes/target proteins/genes are the main inroads into sphingolipid research. Two experimental approaches are illustrated which aim to validate and expand discoveries related to lipids or enzymes/proteins/genes. The various steps in the two approaches have been discussed in the main text and illustrated in various Figures throughout the manuscript, therefore references to these have been included to better orient the reader.

Toward the end of the last century, a new paradigm emerged for the roles of sphingolipids in biologic function and disease: not just complex (glyco)sphingolipids are important, but also, the component parts and metabolic intermediates. That necessitated development of new tools that can analyze essentially all the sphingolipids in biological materials, and many new discoveries have already resulted from those analyses. As the tools for lipidomics analysis are becoming more sophisticated and generating ever larger data sets, the toughest challenge is how to interpret them. The field is also rapidly expanding, partly because of “newcomers” who come across sphingolipids and their enzymes as a result of using various omics approaches. These newcomers may initially find the field daunting. To streamline and assist the decision-making process on where to go next, in Figure 9, we have put together a workflow illustrating the two main experimental paths. As discussed in the section “Approaches to unravel sphingolipid metabolism and function,” two approaches can be adopted to confirm and expand initial sphingolipid discoveries: a lipid centric one if the entry point are results from metabolomics/lipidomics studies (from untargeted analysis) and where the goal is to define the functional lipid(s). An enzyme/protein centric approach is pursued if the entry point are changes of sphingolipid proteins/enzymes/genes (from unbiased screens) and where the goal is to define the function of the enzyme. The two approaches intersect when, building on validated changes in the lipid levels, one aims to discover the source of the changes or vice versa, when one wants to link changes of a specific enzyme/protein with specific sphingolipid profile and biological functions. The tighter the concordance between the approaches, the more rigorous the study will become.

At this juncture in “sphingolipidology,” we are witnessing rapid advances that are tackling many of the challenges raised in the section “Complexities in the study of sphingolipids.” While the methodologies and biologic discoveries have galloped in the past 3-4 decades, the key challenges moving forward stem from a rather incomplete understanding of the myriad biochemical and molecular mechanisms by which the sphingolipid pathways are regulated (by both feedback mechanisms within the pathway and external inputs) and the mechanisms by which the various bioactive sphingolipids exert their functions through direct and indirect targets. From a methodological point of view, the integration of lipidomics with metabolomics, transcriptomics and proteomics requires deep learning capacity not yet broadly available. Furthermore, as we begin to move in the single cell sphingolipidomics space (237, 280), we are faced with the limited sensitivity of sphingolipid measurements particularly for bioactive sphingolipids, which restricts the depth of the information; this becomes an even greater challenge if we seek to apply to single cell analysis some of the techniques discussed, such as labeling, where the pools of targeted sphingolipids are much smaller. At the cell biologic level, a key challenge moving forward is to zero in on compartment-specific functions and regulation.

This review has covered some of the important concepts plus specific details that are useful to be aware of in thinking about sphingolipidomic data whether one is learning about them for the first time or already has expertise and wondering about whether they have overlooked important aspects. Accordingly, not all topics have been covered nor every bibliographic reference cited; however, the authors hope that this overview makes it easier for readers to get into the literature and follow their findings wherever they lead.

## Data availability

Not applicable.

## Conflict of interest

The authors declare that they have no conflicts of interest with the contents of this article.
